# Signaling network analysis reveals fostamatinib as a potential drug to control platelet hyperactivation during SARS-CoV-2 infection

**DOI:** 10.3389/fimmu.2023.1285345

**Published:** 2023-12-21

**Authors:** Özge Osmanoglu, Shishir K. Gupta, Anna Almasi, Seray Yagci, Mugdha Srivastava, Gabriel H. M. Araujo, Zoltan Nagy, Johannes Balkenhol, Thomas Dandekar

**Affiliations:** ^1^ Functional Genomics & Systems Biology Group, Department of Bioinformatics, Biocenter, University of Wuerzburg, Wuerzburg, Germany; ^2^ Evolutionary Genomics Group, Center for Computational and Theoretical Biology, University of Würzburg, Würzburg, Germany; ^3^ Institute of Botany, Heinrich Heine University, Düsseldorf, Germany; ^4^ Core Unit Systems Medicine, University of Wuerzburg, Wuerzburg, Germany; ^5^ Algorithmic Bioinformatics, Department of Computer Science, Heinrich Heine University, Düsseldorf, Germany; ^6^ University Hospital Würzburg, Institute of Experimental Biomedicine, Würzburg, Germany; ^7^ Chair of Molecular Microscopy, Rudolf Virchow Center for Integrative and Translation Bioimaging, University of Würzburg, Würzburg, Germany; ^8^ European Molecular Biology Laboratory (EMBL) Heidelberg, BioComputing Unit, Heidelberg, Germany

**Keywords:** signaling network, controllability, platelet, SARS-CoV-2, fostamatinib, drug repurposing, COVID-19

## Abstract

**Introduction:**

Pro-thrombotic events are one of the prevalent causes of intensive care unit (ICU) admissions among COVID-19 patients, although the signaling events in the stimulated platelets are still unclear.

**Methods:**

We conducted a comparative analysis of platelet transcriptome data from healthy donors, ICU, and non-ICU COVID-19 patients to elucidate these mechanisms. To surpass previous analyses, we constructed models of involved networks and control cascades by integrating a global human signaling network with transcriptome data. We investigated the control of platelet hyperactivation and the specific proteins involved.

**Results:**

Our study revealed that control of the platelet network in ICU patients is significantly higher than in non-ICU patients. Non-ICU patients require control over fewer proteins for managing platelet hyperactivity compared to ICU patients. Identification of indispensable proteins highlighted key subnetworks, that are targetable for system control in COVID-19-related platelet hyperactivity. We scrutinized FDA-approved drugs targeting indispensable proteins and identified fostamatinib as a potent candidate for preventing thrombosis in COVID-19 patients.

**Discussion:**

Our findings shed light on how SARS-CoV-2 efficiently affects host platelets by targeting indispensable and critical proteins involved in the control of platelet activity. We evaluated several drugs for specific control of platelet hyperactivity in ICU patients suffering from platelet hyperactivation. The focus of our approach is repurposing existing drugs for optimal control over the signaling network responsible for platelet hyperactivity in COVID-19 patients. Our study offers specific pharmacological recommendations, with drug prioritization tailored to the distinct network states observed in each patient condition. Interactive networks and detailed results can be accessed at https://fostamatinib.bioinfo-wuerz.eu/.

## 1 Introduction

COVID-19 caused by SARS-CoV-2 has caused more than 769 million infections and 6.9 million death cases until August 2023, as per World Health Organization (WHO). It is well known that SARS-CoV-2 can cause pneumonia and acute respiratory distress syndrome (ARDS), as well as several extrapulmonary manifestations. Recent reports showed that it can also lead to multiorgan failure and thrombosis, including myocardial infarction and ischemic stroke (Connors & Levy, 2020). In addition, while only 1.3% of non-COVID-19 intensive care unit (ICU) patients develop thrombotic issues, the cumulative incidence of thrombotic complications for ICU admitted COVID-19 patients was 49% (1). According to large-scale studies, on admission, 18.8% to 36.2% of patients (2, 3) exhibit thrombocytopenia. These studies show the importance of platelet studies in COVID-19 patients (4), especially in patients admitted to the intensive care unit (ICU) who are at the highest risk of thrombosis. Platelets have an important role in maintaining primary hemostasis and blood flow within the vessel. Following the injury of a vessel, the platelets circulating in the blood become activated, resulting in adhesion in the exposed extracellular matrix and the formation and consolidation of a thrombus. Following adhesion, signal transduction leads to platelet activation, cytoskeletal changes (and change into dendritic shape), and the activation of integrins that support adhesion and aggregation of new platelets (5). During infection with a virus, activated platelets adhere to the sub-endothelium, and their hyperactivity causes thrombus formation, which can trigger arterial ischemia and even pulmonary embolisms. Like many other viruses such as the influenza virus (H1N1), human cytomegalovirus (HCMV) (6), immunodeficiency virus (HIV) (7), dengue virus (8, 9), hepatitis C virus (HCV) (10) and Ebola (11) and many other viruses (12), SARS-CoV-2 (13–16) can also directly lead to platelet hyperactivity.

Zhang et al. (2020) showed that platelets from COVID-19 patients express ACE2 and TMPRSS2, and the Spike protein of SARS-CoV-2 can bind to platelet ACE2 and increase platelet activation (13). In addition, Shen et al. (2021) revealed platelets might take up SARS-CoV-2 mRNA independent of the receptor of SARS-CoV-2, ACE2 (17). Further studies showed hyperactivity of platelets in COVID-19 infection (18, 19), although other studies published contradicting results with hypoactive platelets (20). Manne et al. (2020) compared RNA-seq data from COVID-19 patients and healthy individuals, revealing differences in gene expression related to protein ubiquitination, antigen presentation, and mitochondrial function. COVID-19 patients had increased P-selectin expression in resting platelets and upon activation. Platelet-neutrophil, -monocyte, and -T-cell aggregates were significantly higher in COVID-19 patients, along with increased platelet aggregation and spreading of fibrinogen and collagen. These changes were linked to heightened MAPK pathway activation and thromboxane generation (19). Recently, Weiss et al. (2023) showed platelet hypoactivity caused by reduced GPIIb/IIIa activation but still procoagulant and more adhesive platelet function in COVID-19 (21), while Denorme et al. observed less procoagulant platelet formation in COVID‐19 patients (22). Despite the recent advances, it is not known to a full extent how the signaling in platelets operates during the SARS-CoV-2 infection. Which proteins in platelets can be used to control cellular output during SARS-CoV-2 infection or platelet hyperactivation under septic conditions in general? To answer these questions, we first modeled context-based signaling networks of platelets of COVID-19 non-ICU and ICU patients, then applied control theory (23) to analyze the networks. In control theory, the driver nodes (genes/proteins) are defined as the minimum number of inputs that can steer the system from any initial state to any final state in finite time (24). We further used the node classification scheme proposed by Vinayagam et al. (2016), i.e. indispensable, neutral, and dispensable (25), and Jia et al. (2013), i.e. critical, intermittent, and redundant (26) to classify the driver proteins in platelet networks.

Our methodology is focused on identifying the most suitable pharmaceutical intervention for specific severe network dysregulation, such as platelet hyperactivity in sepsis, with particular emphasis on the context of COVID-19. The key question for medical professionals, particularly in intensive care units, is how to effectively treat the patient with the available drugs. To answer this key point, we first reanalyzed the RNA-seq data (19) of platelets isolated from healthy donors and SARS-CoV-2–infected non-ICU and ICU patients. We further reconstructed the context-specific signaling network of ICU and non-ICU platelets. By applying the controllability theory, we classified the network nodes and further connected the important nodes with drug targets present in the DrugBank database (27). Based on our network analysis, we repurpose fostamatinib as a highly potent drug that can be used to control platelet hyperactivity in COVID-19 patients. Overall, our study contributes to the understanding of platelet biology during COVID-19.

## 2 Materials and methods

### 2.1 Data collection and differential expression analysis

We reanalyzed raw original RNA-seq data [PRJNA634489; published by Manne et al.2020 (19)] of platelets isolated from healthy donors, SARS-CoV-2–infected non-ICU and ICU patients. The quality of Illumina reads was assessed by using FastQC (28). The quality-filtered reads were mapped against the human genome reference (GRCh38.p19) using STAR (29). FeatureCounts was used for assigning sequence reads to genomic features (30). Following standard normalization procedures and reducing batch effects, the BioConductor package DESeq2 (31) was used to identify genes significantly differentially expressed between the three conditions (healthy donors, non-ICU COVID-19 patients, ICU COVID-19 patients). Genes with fold change ≥ |+/−1.5| and padj< 0.05 were considered significantly differentially expressed.

### 2.2 Platelet gene annotation

We used Gene Ontology Resource (32) to annotate the known platelet genes. GO categories platelet activation (GO:0030168), negative regulation of platelet activation (GO:0010544), positive regulation of platelet activation (GO:0010572), platelet aggregation (GO:0070527), negative regulation of platelet aggregation (GO:0090331), positive regulation of platelet aggregation (GO:1901731), platelet degranulation (GO:0002576), blood coagulation (GO:0007596), negative regulation of blood coagulation (GO:0030195) and positive regulation of blood coagulation (GO:0030194) were used to annotate differentially expressed genes. For simplification, we termed proteins coded by these genes as Platelet Gene Set (PGS).

### 2.3 Signaling network reconstructions

To model the active signaling networks for non-ICU and ICU patient platelets, we used SPAGI (33) and ViralLink (34) workflows. In brief, we used the log2 fold change from healthy donors versus SARS-CoV-2–infected non-ICU and from healthy donors versus SARS-CoV-2–infected ICU patients in both the workflows to construct the signaling networks which we named as non-ICU-s and ICU-s (for SPAGI networks) and non-ICU-v and ICU-v (for ViralLink networks) respectively. SPAGI uses its own classification of signaling network components to identify the signaling subnetworks that begin from cellular receptors by integrating directed protein-protein interaction network and gene expression data (33). ViralLink reconstructs the signaling pathways starting from intracellular SARS-CoV-2 proteins, leading to the host cell receptor, signaling components, and downstream differentially expressed genes (34). We wanted to add repurposed drugs at the top layer of the reconstructed signaling network after controllability analysis. Therefore, we removed the virus protein layer from the ViralLink-based non-ICU-v and ICU-v networks and added drugs. For further algorithmic details of the applied network building workflows, we recommend the readers to consider references (33) and (34).

### 2.4 Integrated network

We combined the non-ICU-s and non-ICU-v to construct the integrated ‘non-ICU-sv’ network. After the merging, we deleted five nodes (TYRO3, CLK3, CLK2, ATR, and RFX3) that were disconnected from the largest component of the integrated network. Similarly, we integrated ICU-s and ICU-v to construct the integrated ‘ICU-sv’ network. Merge function of Cytoscape (35) was used for the network integration. We noted that heparanase (HPSE) was absent in our integrated network, although the high activity of HPSE (36) has been reported in platelets during sepsis (37) and in COVID-19 patients (36). To include HPSE in both the integrated networks, we added three additional literature-curated activation interactions that connects EGR1, RELA (38), and NFKB1 (39) to HPSE.

### 2.5 Controllability analysis and node classification

To identify the driver nodes in the reconstructed active networks, we implemented the concept of controllability and used Minimum Dominating Set (MDS) (40, 41), a graph theory-based approach for network analysis (35, 42). Non-linear systems, such as complex biological networks, can be efficiently managed using the MDS method (43). Since, for a given biological network, the MDS is not unique, we utilized the node classification schemes recommended by Vinayagam et al. (2016), *i.e.*, indispensable, neutral, and dispensable (25) and Jia et al. (2013), *i.e.*, critical, intermittent, and redundant (26) to identify the important driver nodes. Since the indispensable nodes are evolutionary conserved, essential, and act as key players for healthy to disease transition (25), we further checked the network for indispensable proteins, which are first to third downstream neighbors of critical proteins and show significant changes in their expression in the corresponding condition and are drug targets. We filtered indispensable nodes down to these nodes and named them filt-ind.

### 2.6 Druggable network node identification

Food and Drug Administration (FDA) approved drugs, and their target information was collected from the DrugBank database (27). Thromboinflammation targeting drugs and their target information were collected from a recent review (44). We further examined the collected drug’s interaction with the proteins that have independently been classified as indispensable including the filt-ind nodes in non-ICU-sv and ICU-sv networks.

### 2.7 High-scoring path calculations

The Transcripts Per Kilobase Million (TPM) values for all genes were mapped onto the constructed non-ICU-sv and ICU-sv networks in the form of node and edge weights.

The node weight *N_i_* of gene *I* was given as


(1) $$N_{i(DH)}=\frac{\sum \frac{TPM_{i(D)}}{n}+1}{\sum \frac{TPM_{i(H)}}{n}+1}$$

where *TPM_i(D)_* represents the TPM value of gene *i* in condition *D* (disease), *n* represents the number of samples, and *TPM_i(H)_* represents the TPM value of gene *i* in condition *H* (healthy).

The edge weight *W_ij_* between gene i and gene j was computed as


(2) $$W_{ij(DH)}=Inverse\sqrt{N_{i}\times N_{j}}$$

A lower edge weight is indicative of an active edge, wherein the interacting nodes have high relative fold changes in two conditions.

The calculated weights were used to convert non-ICU-sv and ICU-sv into weighted networks. Further, filt-ind proteins, which were identified as known drugs modulated proteins, were kept as ‘source’ nodes, and along with classical platelet markers, upregulated platelet proteins PGS were kept as ‘target’ nodes. To identify the meaningful control space, we attempted to connect each source with all the target nodes using the PathLinker (45) application of Cytoscape (35). For this PathLinker analysis, the parameter (k), which indicates the number of paths, was set to 221 for ICU and 70 for non-ICU networks (the number of filt-ind multiplied by the number of upregulated platelet proteins in the network). Additionally, for the ICU network, we computed the lowest-cost paths between each source and target separately by setting k=1 and selecting the PathLinker additive edge weights option. Here, the path cost indicates the sum of edge weights.

### 2.8 Experimental validation: platelet preparation

Wild type C57BL/6 mice were euthanized under isoflurane anesthesia and then immediately bled into heparin (20 U/ml, Ratiopharm), and blood was washed twice using Tyrode-HEPES buffer. Platelet-rich plasma (PRP) was supplemented with 2 μl/ml apyrase (0.02 U/ml; A6410, Sigma-Aldrich) and 5 μl/ml PGI2 (0.1 μg/ml; P6188, Sigma-Aldrich) and platelets were pelleted by centrifugation for 5 min at 2800 g, washed once with Tyrode-HEPES buffer (134 mM NaCl, 0.34 mM Na2HPO4, 2.9 mM KCl, 12 mM NaHCO3, 5 mM HEPES, 5 mM glucose, 0.35% BSA, pH 7.4) containing 2 μl/ml apyrase and allowed to rest for 30 min prior to experiments.

### 2.9 Experimental validation: immunoblotting

For testing the effect of R406 (active metabolite of fostamatinib) on platelet signaling, washed platelets adjusted to a concentration of 5 x 10^8^/ml were pre-incubated with 1 µM R406 or 0.1% DMSO *in vitro* for 10 minutes. Afterwards, platelets were either left unstimulated or were incubated with CRP (10 µg/ml) for 90s under stirring conditions (1200 rpm, 37°C). Samples were immediately lysed in IP buffer (15 mM TRIS HCl, 155 mM NaCl, 1mM EDTA. 0.005% NaN3, supplemented with 2% NP-40) containing 1x Halt protease and phosphatase inhibitors for 10 min on ice. Samples were centrifuged for 10 min at 14000 rpm at 4°C, and the supernatant was kept at -80°C until analysis. For immunoblotting, samples were mixed with 4x reducing Laemmli buffer and boiled for 5 min at 95°C. Denatured proteins were separated by SDS-PAGE and blotted onto PVDF membranes. Membranes were probed for Syk p-Y525/526 (#2711), SFK p-Y416 (#6943), and GAPDH (#2118). All antibodies were purchased from Cell Signaling Technology. Bound antibodies were detected using horseradish-peroxidase-conjugated secondary antibodies and enhanced chemiluminescence solution (JM-K820- 500, MoBiTec). Images were acquired with an Amersham Image 680 (GE Healthcare).

## 3 Results

### 3.1 Platelet transcriptome profile

**Figure 1:**
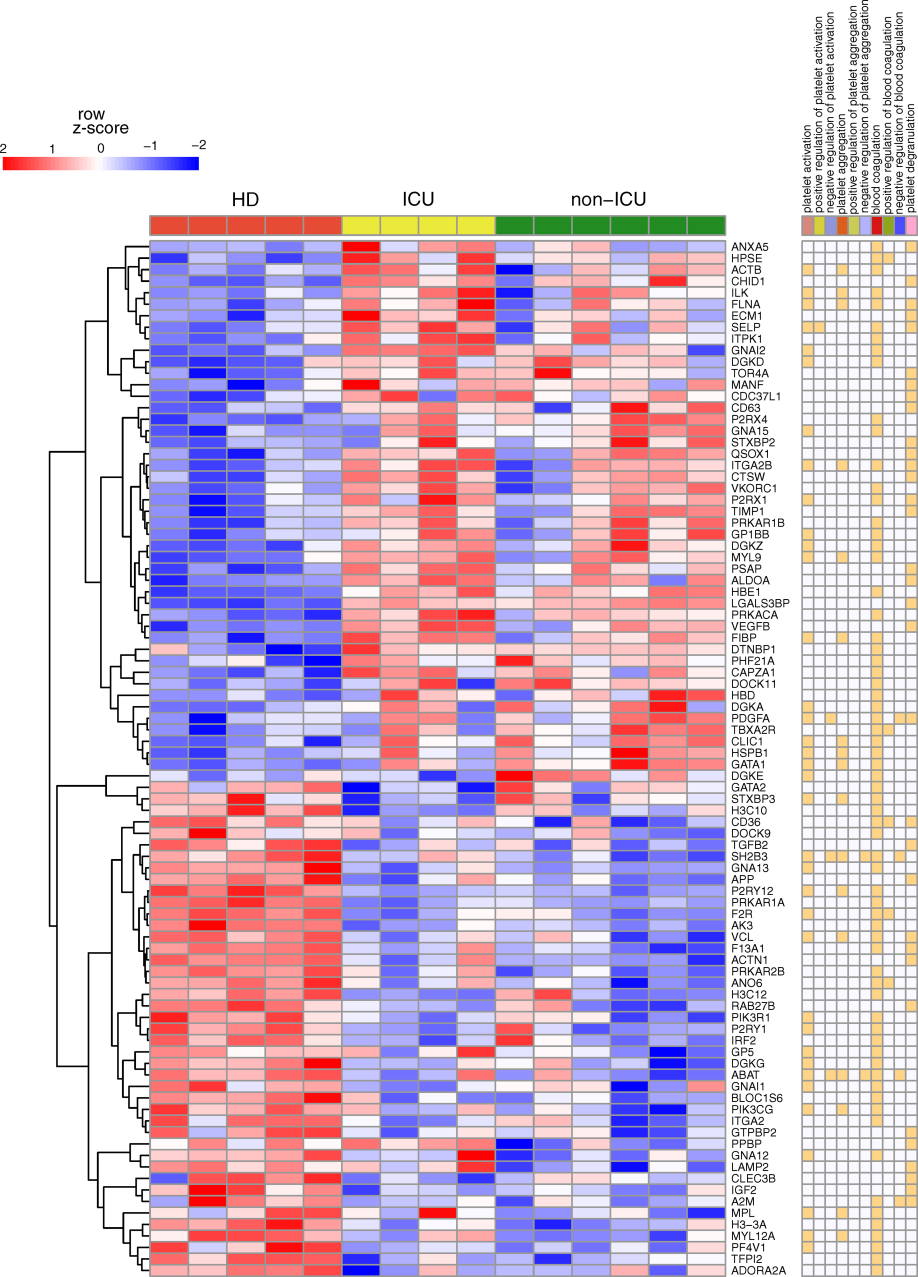
Key gene expression differences for non-ICU and ICU patients in platelets. Heatmap of RNA-Seq expression z-scores computed for genes classified under Platelet Gene Set (PGS) that are differentially expressed (p adj< 0.05, |log 2 (foldchange)| ≥ 0.58) between healthy donors (HD) vs. ICU or in HD vs. non-ICU comparisons. In the right panel, annotations of genes are given. If the genes are classified under the mentioned category, it is marked with orange color.

SARS-CoV-2 infection dysregulates platelet function, which contributes to COVID-19 pathophysiology. The significance of platelets in viral infection–mediated thrombosis has previously been established (46). Using platelet RNA sequencing, Manne and colleagues (2020) revealed platelets contribute to thrombosis formation in SARS-CoV-2 infection (19). However, though this study provided unique raw data (PRJNA634489), the mechanisms underlying platelet activation in COVID-19 patients remained obscure. We used network biology and RNA-Seq integration to explore platelet signaling in COVID-19 patients. We reanalyzed the RNA-seq data and found 2,900 genes that were differentially expressed in non-ICU patients compared to healthy donors. In comparison, 2,254 genes were differentially expressed in ICU patients compared to healthy donors. The numbers are very similar to Manne and colleagues’ original study, and overall, there was good overlap in analyzed genes in which 3090 and 2256 differentially expressed genes were found in non-ICU and ICU patients, respectively (details in **Supplementary Tables 1–3**; differences were only from normalization and cut-offs, compare the two methods sections). However, starting from this gene expression analysis, we did a detailed and new network analysis, starting from pathway enrichment: among the known annotated genes in the Gene Ontology (GO) category’ positive regulation of platelet activation’ (GO:0010572), only ICU patients showed significantly higher expression of SELP (P-selectin). In contrast to the study of Hottz and colleagues (47), increased CD63 expression was found in non-ICU patients but not in ICU patients. As shown in **Figure 1**, compared with healthy controls, COVID-19 patients showed differential expression of many genes involved in platelet activation and blood coagulation.

**Figure 2:**
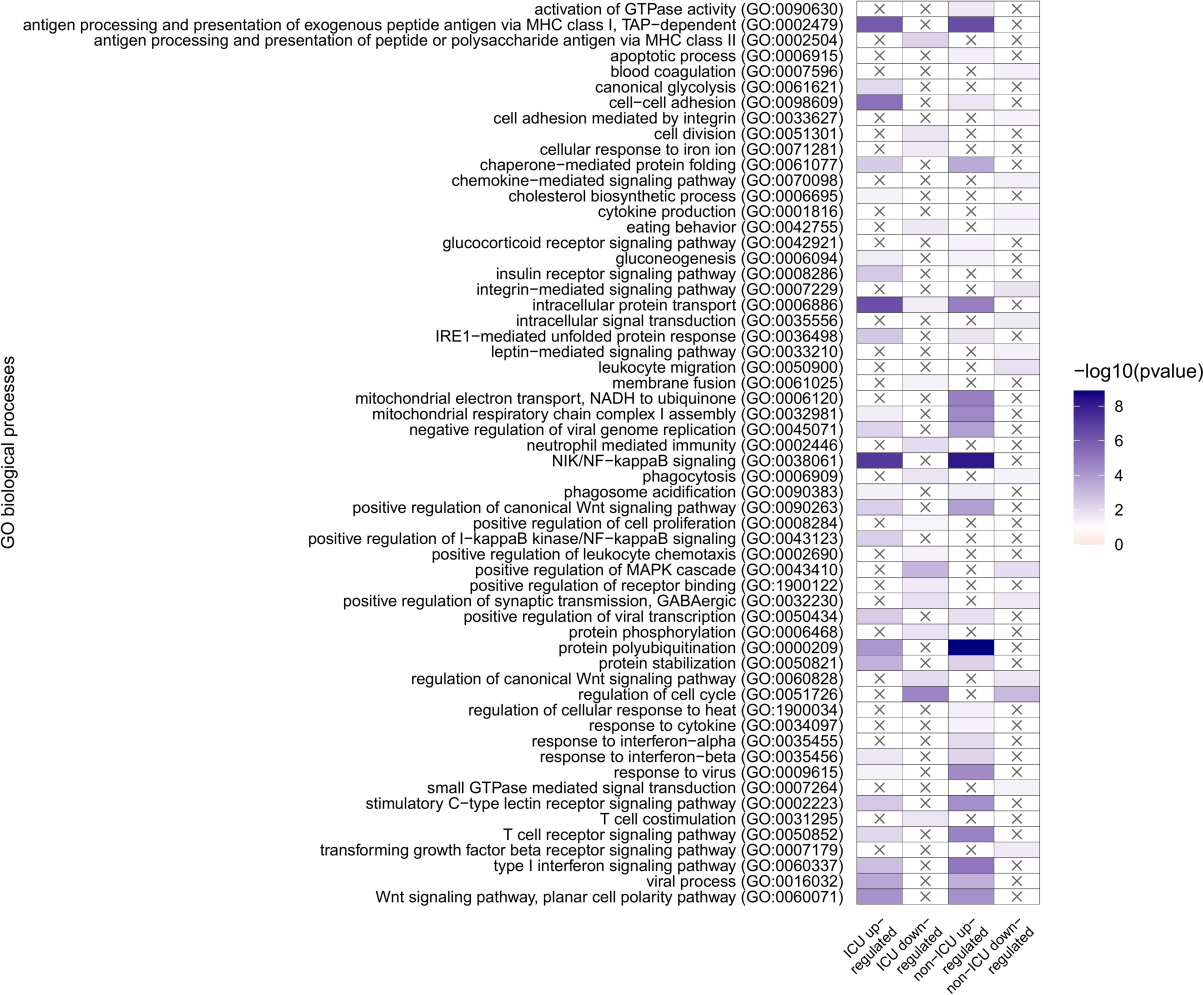
GO overrepresentation analysis of genes uniquely and differentially expressed in non-ICU or ICU patients. Overrepresented pathways are displayed in four groups: ICU upregulated and downregulated, and non-ICU upregulated and downregulated.

GO analysis of genes uniquely and differentially expressed in non-ICU or ICU patients identified overrepresentation of NF-kappaB signaling pathway (GO:0038061) and cell adhesion (GO:0098609) in both non-ICU and ICU upregulated genes (**Figure 2**). Furthermore, upregulated genes in both conditions were enriched in protein folding and stability-related processes, some metabolic processes, several signaling pathways and, more importantly, immunity-related processes, including MHC class I, antigen presentation (GO:0002479), several virus responses, and signaling processes initiated by interferon-β and T cell receptor (GO:0035456, GO:0050852).

Commonly overrepresented processes in ICU and non-ICU downregulated gene sets are comparably lower in number and include processes like phagocytosis (GO:0006909), and cell cycle (GO:0051726); and signaling pathways such as MAPK cascade (GO:0043410 canonical Wnt signaling pathway (GO:0060828).

On the other hand, overrepresentation of positive regulation of I−kappaB kinase/NF−kappaB signaling (GO:0043123) is specific to ICU upregulated genes, while the ICU downregulated gene set has a specific overrepresentation of antigen processing and presentation of peptide or polysaccharide antigen via MHC class II (GO:0002504), neutrophil-mediated immunity (GO:0002446), positive regulation of leukocyte chemotaxis (GO:0002690), and T cell co-stimulation (GO:0031295). Upregulated genes from non-ICU patients show a specific overrepresentation of activation of GTPase activity (GO:0090630), response to cytokine (GO:0034097), and response to interferon−alpha (GO:0035455, while downregulation of blood coagulation (GO:0007596), cell adhesion mediated by integrin (GO:0033627), chemokine−mediated signaling pathway (GO:0070098), cytokine production (GO:0001816), integrin−mediated signaling pathway (GO:0007229), intracellular signal transduction (GO:0035556), leptin−mediated signaling pathway (GO:0033210), leukocyte migration (GO:0050900), small GTPase mediated signal transduction (GO:0007264), and transforming growth factor-beta receptor signaling pathway (GO:0007179) are only observed in the non-ICU patients.

Looking at the overrepresented GO categories, we have found processes involved mainly in immune-related processes, including antigen presentation, NFKB signaling, interferon responses, responses to virus, phagocytic responses, and neutrophil-mediated immunity, along with general leukocyte responses, chemokine and cytokine-mediated signaling pathways. In non-ICU patients, we even observed an overrepresentation of blood coagulation as a downregulated process, and in ICU patients, no direct platelet process was overrepresented; instead, it reflected the platelets’ contribution to the activation of several immune responses from both innate and adaptive immune cells, which can be the result of hyperactive platelets. Indeed, similar genes were found in COVID-19 to induces a hyperactive phenotype in circulating platelets (16). Moreover similar platelet biomarkers as from our GO analysis were already associated with coagulation dysfunction (15) and with the composite outcome of thrombosis or death (14).

### 3.2 Platelet signaling networks

We created the final directed networks for both ICU and non-ICU conditions by combining networks from SPAGI and ViralLink. After final modifications (see details in material and methods), the ICU final network has 1136 nodes and 7185 edges, while the non-ICU network consists of 937 nodes and 4672 edges. These two final networks share 4227 edges and 820 common nodes (**Supplementary Figure 1**), including platelet activation genes, such as NFKB1 (nuclear factor kappa B subunit 1), RELA (RELA proto-oncogene, NF-kB subunit), and HPSE (heparanase). We added edge weights to both networks to represent the combined regulation of the two nodes interacting. The edge weights were calculated using the fold changes of the interacting nodes in each condition (Equations 1 and 2, **Supplementary Tables 4, 5**). A lower edge weight signifies a higher regulation (positive or negative) of the interaction (see details in material and methods).

The ICU network is more connected with ~6 edges per node compared to the non-ICU network with ~5 edges per node. Regarding connectivity of individual nodes, the ICU network has a higher average number of neighbors of 12.338 than the non-ICU network with 9.823. The characteristic path length, which describes the shortest path between two nodes, is shorter in ICU, 3.987 than in non-ICU, 4.114. The number of multi-edge node pairs shows how often neighboring nodes are linked by more than one edge. This value is 175 at ICU and only 73 at non-ICU and indicates topological differences between the two networks (**Supplementary Tables 6, 7**).

### 3.3 Control robustness: non-ICU-sv and ICU-sv networks

We used two approaches to determine controllability. In the first approach implemented by Vinayagam et al. (25), we define MDS (minimum set of driver nodes) as a set of nodes through which we can achieve control of the whole network. When a node is eliminated and the size of the Minimum Dominating Set (MDS) expands, it signifies the node’s indispensability. Its removal would necessitate exerting control or influence over a greater number of nodes to bring about state changes (e.g., PRKCD (protein kinase C delta), RAC1 (Rac family small GTPase 1), or SRC (SRC proto-oncogene, non-receptor tyrosine kinase); see https://fostamatinib.bioinfo-wuerz.eu/icu.html). If altering the MDS size is not observed, the node holds a neutral role. However, if the MDS size diminishes, the node becomes dispensable. An alternative approach stems from Ravindran et al. (48), employing two distinct techniques to pinpoint driver nodes (1): the minimum dominating set (MDS) and (2) the maximum matching approach. In our study, we also adopted the MDS methodology, which allowed us to classify nodes into three categories: critical (ITGB1 (integrin subunit beta 1), CSNK2A2 (casein kinase 2 alpha 2), IRF3 (interferon regulatory factor 3), encompassing those present in all conceivable MDS; intermittent, signifying inclusion in some MDS; and redundant, relating to nodes absent in any MDS configuration. It is important to note that achieving comprehensive network control can yield various MDS configurations, as demonstrated by Liu et al. (49).

In the non-ICU network, 11.63% of nodes were indispensable, 32.87% were neutral, and 55.5% were dispensable. At the same time, 11.52% were critical, 56.46% were intermittent, and 32.02% were redundant. We observed no overlap between critical and indispensable nodes as well as no overlap between redundant and dispensable nodes (**Supplementary Table 8**).

In the ICU network, 16.55% of nodes were indispensable, 38.64% were neutral, and 44.81% were dispensable. At the same time, 10.56% were critical, 56.69% were intermittent, and 32.75% were redundant. Similarly, no overlap between critical and indispensable and between redundant and dispensable nodes was observed (**Supplementary Table 9**).

Furthermore, we observed differences in the degree (number of connections) distributions of indispensable nodes compared to neutral and dispensable nodes in both networks (**Supplementary Figure 2**). While neutral and dispensable nodes are heavily distributed in lower in- and outdegree numbers, indispensable nodes are distributed over a wider range. We also found that indispensable nodes have higher in- and outdegrees than neutral and dispensable nodes on average. A comparison of both networks showed an increase in the average indegree of dispensable and indispensable nodes in the ICU network, while neutral nodes decreased in their indegrees (**Supplementary Figures 2A, C**). On the other hand, the average outdegree increased for neutral and indispensable nodes in the ICU network compared to the non-ICU network, while dispensable nodes decreased in their outdegree (**Supplementary Figures 2B, D**). In summary, indispensable nodes have the highest number of incoming and outgoing interactions in both conditions. At the same time, these results show a generally increasing pattern in the number of interactions for all node types from non-ICU to ICU (with the two exceptions mentioned above).

Next, we performed the same comparison on nodes classified according to their controllability (**Supplementary Figure 3**). Here, as expected from nodes that are classified as driver nodes in all MDS, we found that critical nodes have an average indegree of 0 in both conditions (**Supplementary Figures 3A, C**). They also have the lowest number of outgoing interactions compared to the non-critical nodes (**Supplementary Figures 3B, D**). On the other hand, redundant nodes, which do not act as drivers in any of the MDS, have the highest average in- and outdegree in both networks, followed by intermittent nodes [same average outdegree as critical nodes in the non-ICU network (**Supplementary Figure 3B**)]. We also observed an increase in average indegree and outdegree of intermittent and redundant nodes during the transition from the non-ICU to the ICU network, while critical nodes showed no change in indegree and a decrease in average outdegree. These results show the opposing nature of criticality to indispensability in the way they react to the change in the control space with the non-ICU to ICU transition.

### 3.4 Understanding non-ICU to ICU state transition using network controllability

**Figure 3:**
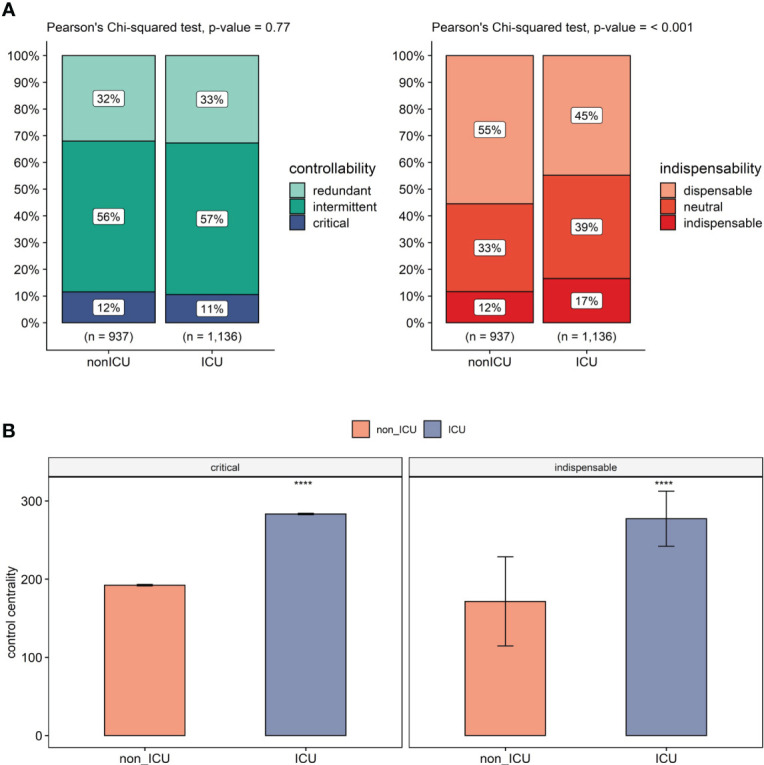
Controllability in platelets comparing non-ICU and ICU. **(A)** Change in controllability (left) and dispensability (right) classification in non-ICU to ICU transition. n: total number of nodes in each network. **(B)** Comparison of control centrality values of critical (left), indispensable nodes (right) in non-ICU and ICU conditions. This shows a clear difference in control centrality for important control nodes (e.g. Src, Syk; details in **Supplementary Tables 8, 9**) of platelets in the ICU patients. The statistical comparison of each group is made using the Mann-Whitney U test and is adjusted by Holm correction. Significance levels are given based on adjusted p-values, ****p<= 0.0001.

In the ICU network, the controllability analysis revealed 42 additional critical nodes to the ICU network, while 78 nodes remained in critical status. Furthermore, during the non-ICU to ICU transition, 30 nodes lost their critical status, with 8 becoming intermittent, 6 redundant, and 16 becoming absent in the ICU network altogether. Among the 42 new critical nodes in ICU network, 3 were intermittent, 2 were redundant in non-ICU, and 37 of these new critical nodes were not present in the non-ICU network. Non-ICU to ICU state transition increases the number of critical nodes in total; however, since the whole network is bigger due to the transition, this increase only corresponds to a 0.96% change. On the other hand, even smaller changes occur in intermittent and redundant nodes, with a 0.23% and a 0.73% increase upon transition, respectively (**Figure 3A**). Regarding critical nodes between non-ICU and ICU networks this data indicates a non-significant change in controllability.

On the other hand, the indispensability analysis revealed in the ICU network 111 new indispensable nodes on top of the 77 nodes that did not change their indispensable status. With non-ICU to ICU transition, 32 nodes lost their indispensable status (22 becoming neutral, 4 dispensable, 6 not present). Among 111 new indispensable nodes, 42 were dispensable, 58 were neutral in the non-ICU network, and 11 were not present. Independent increase in the total network size, the transition resulted in a 4.92% increase in indispensable nodes mainly due to the status shift from dispensable nodes that decreased by 10.69%. Neutral nodes also showed a 5.77% increase during non-ICU to ICU transition. Our data shows a significant increase between the non-ICU and ICU networks regarding indispensable nodes (**Figure 3A**).

We next compared the average control centrality (CC) measures of critical and indispensable nodes in non-ICU and ICU networks (**Figure 3B**). Critical nodes had an average control centrality of 192.29, while it increased significantly to 283.27 in the ICU network (p<.001, Mann-Whitney U test). A significant increase in CC was also observed for indispensable nodes from an average of 154.97 to 258.64 (p<.001, Mann-Whitney U test). Moreover, critical nodes showed higher average control centralities than indispensable nodes in both non-ICU and ICU networks (p<.001, Mann-Whitney U test).

Although no change in the annotation of critical nodes was observed upon non-ICU to ICU transition, there is a significant increase in control centrality of critical nodes. The same is observed for indispensable nodes, with an additional increase in the number of indispensable nodes. Control centrality depicts the size of the controllable downstream subnetwork of a driver node (50). For critical nodes, although the proportion of drivers of the network did not change in the ICU state, they had a higher average control centrality, meaning they control a bigger subsystem, i.e., a similar number of critical nodes that achieve a higher power of control on the network. On the other hand, indispensable nodes cover a higher proportion of the ICU network than non-ICU and their control centrality increases. Therefore, the ICU network offers a higher number of targets to obtain a larger control ability on the network. Collectively, our data indicates an increase in controllability during the non-ICU to ICU transition. This is driven by an increase in the size of the controllable downstream signaling of critical nodes and by an increase in both the proportion of indispensable nodes in the network and their control power on the network.

### 3.5 Drug repurposing

One of the major objectives of our study was to identify the drugs that can help control platelet hyperactivity in COVID-19 patients. Therefore, we extracted the subnetworks. We looked at the controllable space of filt-ind nodes, and if some of these nodes lacked paths to connect with platelet proteins, we discarded these nodes for further analysis (Full lists of filt-ind nodes are given in **Supplementary Tables 10**, **11**). This decision was made for simplification. If a source node (referred to as filt-ind) cannot establish any connections with a target node (in this case, platelet proteins) using any possible route, then that starting point node is unlikely to be highly useful for regulating platelet hyperactivation. To refine our notion of effective control, we also disregarded downstream nodes of filt-ind that are not associated with platelet proteins. Essentially, these downstream nodes not linked to PGS don’t require control for our purposes. Subsequently, we removed filt-ind nodes that don’t have any connections to platelet protein nodes through any pathways. By implementing these filtering steps, we managed to decrease the size of our networks and generate smaller subnetworks for analysis.

**Figure 4:**
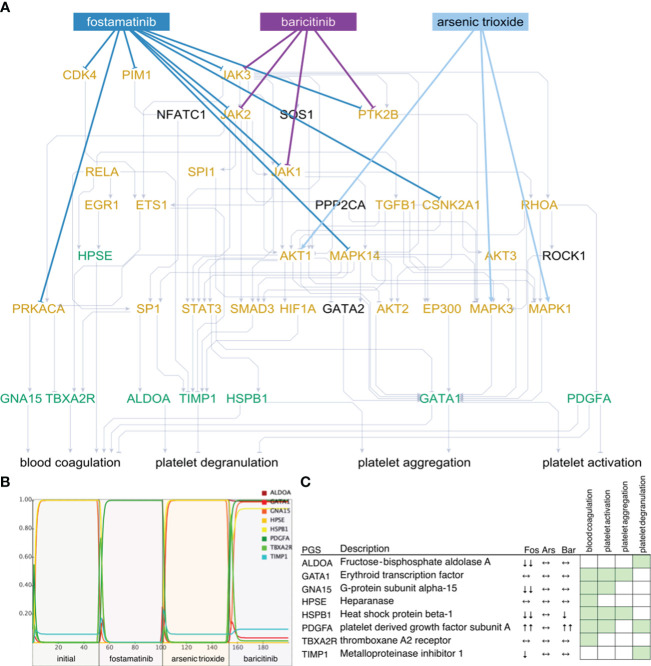
Network state and simulations for non-ICU patients. Subnetworks of controllable space of filt-ind nodes **(A)** targeted by top three drugs: fostamatinib (Fos), arsenic trioxide (Ars), and baricitinib (Bar) in non-ICU network. Simulations **(B)** are summarized for each platelet gene **(C)** and activity change of the platelet genes shown in simulations. Green: platelet gene set (PGS), orange: indispensable nodes.

**Figure 5:**
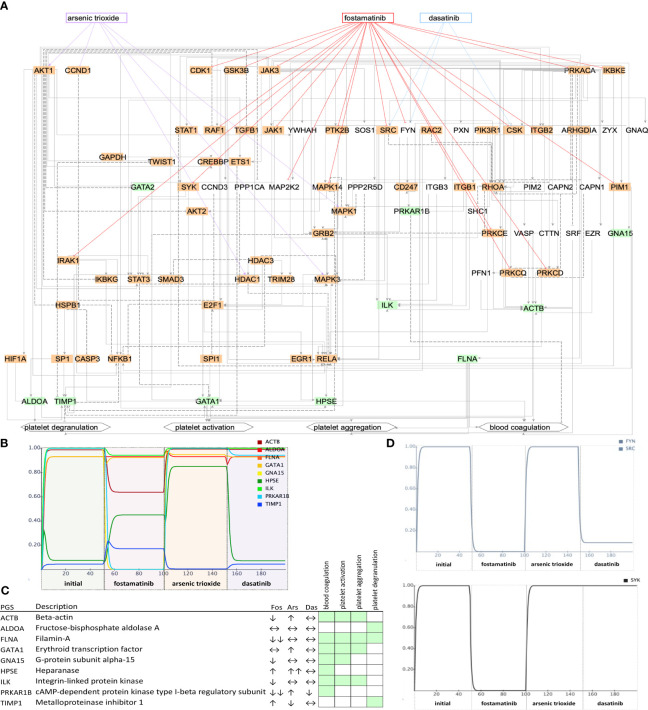
Network state and simulations for ICU patients. Subnetworks of controllable space of filt-ind nodes **(A)** targeted top three drugs: fostamatinib (Fos), arsenic trioxide (Ars), and dasatinib (Das) in ICU network. Simulations **(B)** are summarized for each platelet gene. **(C)** Activity change of the platelet genes **(D)** and of SYK, FYN, and SRC shown in simulations. Green: platelet gene set (PGS), orange: indispensable nodes.

We checked whether these subnetworks could be targeted by FDA-approved drugs in the DrugBank database (27). For the non-ICU network (**Figure 4A**), we have found a total of 29 drugs acting on 15 of the 26 indispensable nodes (9 drugs acting on 5 filt-ind nodes), while for the ICU network, we found 67 drugs controlling 34 of the 52 indispensable nodes (21 drugs controlling 12 filt-ind) nodes, 20 of which are common in both networks [e.g. MAPK1 (mitogen-activated protein kinase 1), TGFB1 (transforming growth factor beta 1), AKT1 (AKT serine/threonine kinase 1), PRKACA (protein kinase cAMP-activated catalytic subunit alpha), RHOA (ras homolog family member A), STAT3 (signal transducer and activator of transcription 3)], whereas only 1 filt-ind node is shared by both networks (JAK3 (Janus kinase 3); see in https://fostamatinib.bioinfo-wuerz.eu/nonicu.html where we give a useful detailed map of individual proteins). The filt-ind nodes JAK3 and CDK4 (cyclin dependent kinase 4; both targeted by 4 drugs) in the non-ICU network and JAK3 in the ICU network (**Figure 5A**) were found to be the most sensitive for manipulation.

Next, we ranked the drugs based on how many indispensable nodes they target with the aim to find the best available drug to treat certain network states observed in COVID-19 patients. In the non- ICU network, we found the top drugs led by fostamatinib with 9 indispensable (4 filt-ind) targets, followed by baricitinib with 4 indispensable (1 filt-ind) targets and arsenic trioxide with 3 indispensable (0 filt-ind) targets, tofacitinib with 3 indispensable (1 filt-ind) and four drugs with 2 indispensable (0 filt-ind except zanubrutinib with 1 filt-ind) targets each: fedratinib, pralsetinib, ruxolitinib, zanubrutinib (**Supplementary Table 12**). In the ICU network, fostamatinib again was found as the top drug with 17 indispensable (6 filt-ind) targets, followed by arsenic trioxide targeting 5 indispensable (2 filt-ind) nodes, dasatinib targeting 3 indispensable (0 filt-ind) targets, baricitinib with 3 indispensable (1 filt-ind), and acetylsalicylic acid, isoprenaline, tofacitinib, vorinostat, cholecystokinin with 2 indispensable (1 filt-ind) targets (**Supplementary Table 13**).

As the top-ranked drug in both networks, we found that fostamatinib, a non-selective SYK (spleen associated tyrosine kinase) pathway inhibitor (51), which is also used in the treatment of chronic immune thrombocytopenia (52), targets around 30% of the indispensable nodes in both ICU and non-ICU networks. Members of the SYK pathway that are targets for fostamatinib were among the indispensable nodes only in the ICU network: SRC (SRC proto-oncogene, non-receptor tyrosine kinase), SYK (Tyrosine-protein kinase SYK) and FYN was neutral in terms of indispensability (FYN proto-oncogene, Src family tyrosine kinase). Other targets that we found only in the ICU network were include CDK1 (cyclin dependent kinase 1), CSK (C-terminal Src kinase), GSK3B (glycogen synthase kinase 3 beta), IKBKE (inhibitor of nuclear factor kappa B kinase subunit epsilon), IRAK1 (interleukin 1 receptor associated kinase 1), PRKCD, PRKCE, PRKCQ (Protein kinase C delta, epsilon, and theta types), and RAF1 (RAF proto-oncogene serine/threonine-protein kinase) (details in https://fostamatinib.bioinfo-wuerz.eu/icu-drugtargets.html). In non-ICU network we found the following nodes to be targeted by fostamatinib: CDK4 (cyclin dependent kinase 4), CSNK2A1 (casein kinase 2 alpha 1), and JAK2 (Janus kinase 2) (details in https://fostamatinib.bioinfo-wuerz.eu/nonicu-drugtargets.html). JAK1 (Janus kinase 1), JAK3 (Janus kinase 3), MAPK14 (Mitogen-activated protein kinase 14), PIM1 (Pim-1 proto-oncogene, serine/threonine kinase), PRKACA (Protein kinase C alpha type), and PTK2B (Protein-tyrosine kinase 2-beta) were common in both networks.

### 3.6 Simulations

Next, we tested the effects of the top-ranked drugs in both non-ICU and ICU networks. We connected the platelet genes (green) to 4 platelet biological processes that were used to construct the platelet proteins: platelet activation, platelet degranulation, platelet aggregation, and blood coagulation. Additionally, we have used the GO terms and literature support for each interaction (**Supplementary Tables 14, 15**).

To investigate their effects on platelet proteins, we simulated the top three drugs, fostamatinib arsenic trioxide and baricitinib, in the non-ICU network (**Figure 4B**). We observed the strongest inhibition of platelet genes ALDOA (aldolase, fructose-bisphosphate A), GNA15 (G protein subunit alpha 15), HSPB1 (heat shock protein family B (small) member 1), and TIMP1 (TIMP metallopeptidase inhibitor 1) by fostamatinib, while arsenic trioxide did not lead to any changes, and baricitinib caused a slight change in the activities of HSPB1 and PDGFA (platelet derived growth factor subunit A, **Figure 4C**). On the other hand, fostamatinib led to a decrease in the activities of ACTB (actin beta), FLNA (filamin A), GNA15, ILK (integrin linked kinase) and PRKAR1B (protein kinase cAMP-dependent type I regulatory subunit beta) in the ICU network, while arsenic trioxide showed mainly stimulating effects and dasatinib did not lead to any changes in activities except PRKAR1B (**Figures 5B, C**). We also showed the inhibiting effect of fostamatinib on SYK, FYN, and SRC in our ICU simulations (**Figure 5D**), and we could confirm this direct inhibiting effect in activated mouse platelets.

### 3.7 Direct experimental validation of fostamatinib effects on platelets

**Figure 6:**
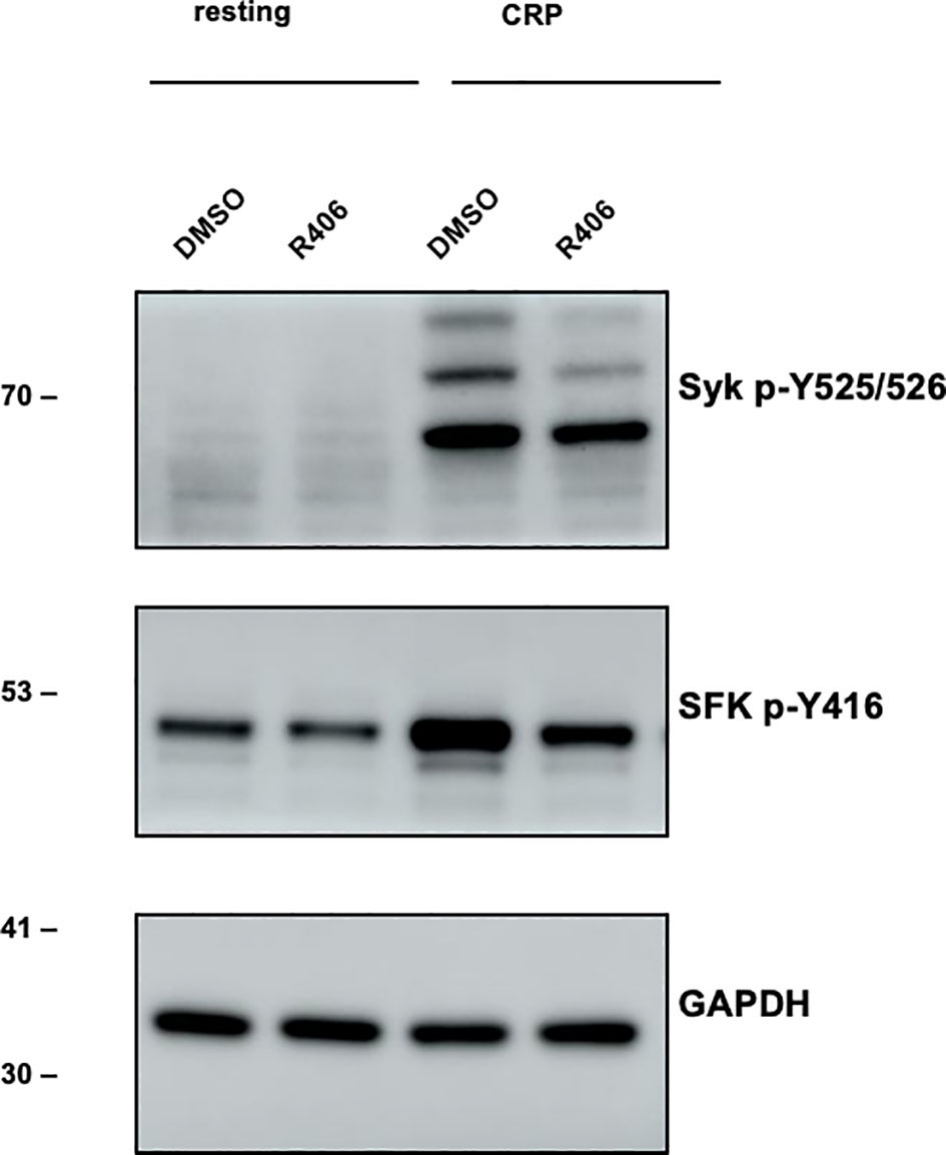
Experimental verification of main fostamatinib targets. Western blot experiment. Left (lane 1,2): Resting platelet. Right (lane 3,4): Stimulation of Syk and SFKs (Src family kinases) in response to CRP (10 µg/ml) [collagen-related peptide; 90s -/+ inhibitor (n=3)]. Lane 3: DMSO control, lane 4: reduced stimulation when incubated with R406 (1 µM) for 10 min. Antibody plots show the results for p-Syk, p-Src family kinase (p-SFK, recognizing p-Src, p-Fyn and p-Lyn) and GAPDH.

To explore the effects of R406, the active moiety of the prodrug fostamatinib processed in the intestine, we applied it directly to washed murine platelets at a concentration of 1 µM (see Methods for preparation and immunoblotting), based on findings from Spalton et al. (2009) (53). Using western blot analyses, we analyzed p-Syk, p-SFKs (which recognizes p-Src, p-Fyn, and p-Lyn), and used GAPDH as a control. Upon stimulation with CRP (10 µg/ml) for 90s, we observed a pronounced activation of Syk and Src family kinases (SFKs). Notably, the activation of these kinases was considerably reduced when platelets were pre-incubated with R406 across all three biological replicates (**Figure 6**).

To extend this promising experimental data on drug action of our top predicted drug further by bioinformatics, including further top candidates, for the ICU network, we also built the lowest-cost paths from each filt-ind node to each platelet node to have an equal contribution of each filt-ind node in the control of platelet genes (**Supplementary Figure 4**) and simulated the effects of three top-ranked drugs. Here, we also confirm the strong inhibitory effect caused by fostamatinib compared to the other two drugs (arsenic trioxide and acetylsalicylic acid).

We also investigated ibrutinib, an inhibitor of Bruton’s tyrosine kinase (BTK) (54), which is following directly after the top scoring drugs (**Supplementary Figure 5**). Ibrutinib has been shown to have a positive effect on a COVID-19 patient with a difficult case of thrombosis (55, 56). Our analysis excluded ibrutinib for two reasons (1): its target BTK was identified as neutral and redundant by our controllability analysis, and (2) with only one known target ibrutinib would fail to be ranked high. Nevertheless, we built its controllable space by finding the shortest paths connecting BTK to platelet proteins. Here, our dynamic simulations showed the strong effect of ibrutinib on platelet genes in ICU (**Supplementary Figure 5**).

In conclusion, our simulations and direct experiments confirm fostamatinib as the best drug on platelet that can be repurposed against thrombotic events observed in COVID-19 patients, especially in ICU patients. Our network analysis shows convincingly that hyperactivated platelets need better control to come to a more balanced state. Including a broad range of platelet kinase modulating drugs we derived several drugs to improve this dangerous ICU condition and overall, according to this analysis and this dataset, fostamatinib was best while only clinical trials can decide which of the top ranked drugs really performs best in clinic.

## 4 Discussion

### 4.1 Understanding platelet dynamics in COVID-19: insights for repurposed drug strategies

In a recent clinical investigation, Manne and colleagues examined platelet involvement in SARS-CoV-2-related thrombosis (19). Among many others, their findings support that SARS-CoV-2 infection is associated with platelet hyperreactivity, which may contribute to COVID-19 pathophysiology. This reflects the critical need for repurposed drugs that can control platelet hyperactivity which prompted our systems biology study. We did a comprehensive bioinformatical analysis of the intricate protein network dynamics - mapping out the hierarchies of protein control, the interplay between various proteins and network nodes, and the identification of indispensable elements within the signal processing cascade – and conduct a rational screening for an optimal drug regimen to address platelet hyperactivity, nevertheless a challenging endeavor.

Utilizing the gene expression data by Manne et al. (19), we created context-specific platelet signaling networks and then searched for FDA-approved drugs that can modulate the network. Analysis of the ICU and non-ICU transcriptome data, we identified genes highlighted genes with heightened expression linked to disease severity. We then looked for the high-importance network nodes as indicated by the controllability method. This method proved to be robust in estimating the extend of the controlled subnetwork (25, 57). Prioritizing indispensable and critical nodes, we reduced the networks to the subnetwork level, given the surplus of terminal nodes with no defined roles in platelet hyperactivity (see materials and methods). Within these subnetworks, we had filt-ind nodes connected with upregulated platelet activity-related genes. Finally, we extended our analysis to identify known drugs capable of fine-tuning this subnetwork to manipulate platelet activity-related genes.

The robustness of our study lies in its thorough examination of the hyperreactive platelet network, enabling drug ranking based on individual patient conditions. We assessed each FDA-approved drug’s network effects comprehensively, considering not only primary targets but also broader impact on related protein structures (all FDA drugs and their targets can be found in **Supplementary Table 16**). Specifically, for the leading drug, fostamatinib, we incorporated its *in vitro* potency (IC50) as experimentally validated by Rolf and colleagues (**Supplementary Table 17**) (58). Recognizing drugs’ broader effect profile, we prioritize the best option, vital for ICU patients. Our research provides insights into selecting the most effective treatment from the available drugs for a given network condition and patient, potentially saving lives in critical settings like ICU.

### 4.2 Network-based approaches to COVID-19 and drug repurposing

Network-based methods are crucial for understanding protein interactions (59), host-pathogen relationships (60), drug mechanisms (61), side-effect discovery (62), novel drug targets, and drug repurposing (63), which is particularly significant during pandemics due to their time and cost efficiency compared to new drug discovery. Cheng et al. developed a human protein-protein interaction network to identify drug-disease indications, side effects, and mechanisms, with high accuracy (63, 64). Recently, several network-based drug repurposing studies have been published for SARS-CoV-2 (65–69) however, none of them have focused on platelets. Gordon et al. identified potential drug targets based on experimentally determined SARS-CoV-2-human protein interactions (66), and Han et al. used computational methods to pinpoint drugs targeting SARS-CoV-2-induced pathways and 20% of the identified drugs were already being tested in COVID-19 clinical trials (67). Jang et al. employed a novel virtual screening strategy for COVID-19, revealing promising drug combinations with antiviral properties and reduced toxicity (65). Zhou et al. screened for drugs based on network proximity analysis between drug targets and coronavirus-associated host proteins (68). More recent drug repurposing studies have revealed several promising antiviral treatment options for COVID-19 based on novel network-based algorithm (70) and on network controllability (71, 72) (more details in **Supplementary Notes**). Nonetheless, prior studies predominantly concentrated on either targeting the virus itself or exploring host-pathogen interactions for potential drug repurposing. In contrast, in our study we place a particular emphasis on the impact of SARS-CoV-2 on platelets to manage the observed platelet hyperactivity, a condition prevalent in both ICU and non-ICU COVID-19 patients.

Our approach initially centers on constructing signaling networks and subsequently determining node importance through controllability analysis. Control theory aids in identifying critical driver nodes, which, when manipulated, can steer the network from its current state to a desired one. Indispensable nodes, crucial for system integrity, raise the minimum number of driver nodes required for control when removed. Additionally, critical nodes, with no incoming connections, appear in every alternative minimum driver set of the system. Endothelial cells as well as leukocytes such as neutrophils and alveolar macrophages (73) play prominent roles in the initiation and continuation of thrombo-inflammation and can be modelled in the same way as done here for platelets. However, specific transcriptome data-sets are required to determine condition-specific system states and design a therapy for ICU versus non-ICU patients looking at endothelial cells or leukocytes. As platelets are pivotal in hyperinflammatory conditions, we focus in this study our attention to platelet hyperactivity. This is an important strength but cell-type wise clear limitation of our study.

Applying control theory to platelet networks revealed following findings: indispensable nodes have the highest number of connections compared to dispensable and neutral nodes in both networks. In contrast, critical nodes positioned upstream in pathways switching the system between different states, have no incoming and the fewest outgoing connections. Unlike indispensable nodes, whose impact on the system intensifies with an increasing number of connections, critical nodes’ significance lies in orchestrating system shifts rather than extensive connections. Despite lower connectivity, critical nodes exhibited higher control centralities, underscoring their potent influence on network controllability, and signaling dynamics. This clarifies the distinct roles of indispensable and critical nodes in steering network behavior.

One of our major findings shows how the transition from non-ICU to ICU can be described in terms of control theory. Although no change in the proportion of critical nodes was observed, we show a 4.92% increase in indispensable nodes in ICU patients, mainly due to the status shift from dispensable to indispensable. Similarly, in ICU patients we have an increase in average control centralities of both critical (by 47.3%) and indispensable nodes (by 66.9%). Furthermore, we observed a greater number of drug targets within the ICU networks (34/81, 42%) when compared to the non-ICU network (15/38, 39,5%). Both findings suggest that the transition to ICU network offers a higher number of targets to obtain a larger control ability on the network and illustrates that platelet signaling and control is quite different in ICU patients.

### 4.3 Drug effects on platelet control: fostamatinib and beyond

Our exploration of FDA-approved drugs aimed to control platelet hyperactivity induced by SARS-CoV-2 infection or septic conditions led to the identification of promising candidates. Fostamatinib emerged as the top-ranked drug across both networks, with an ability to regulate multiple dysregulated proteins, particularly within the ICU network. While the exact mechanism of fostamatinib remains unspecified due to its limited specificity toward SYK, inhibiting other kinases and non-kinase targets at concentrations comparable to those inhibiting SYK (74). Therefore, its effect is based on the inhibition of SYK-dependent and SYK-independent immune signaling pathways (75). Besides its effect on SYK and SFKs, in our results, we found a major effect of fostamatinib on SYK-independent immune signaling pathways.

Notably, arsenic trioxide surfaced as a top-ranked drug in both networks, known for its use for the treatment of acute promyelocytic leukemia (76). Its anti-platelet activity has also been observed mainly via its inhibition of the PLCγ2-PKC-p38 MAPK cascade (77). However, it is more a powerful manipulator of the ICU network, as majority of the targets of arsenic trioxide are activated by the drug, leading to possibly higher platelet activity. Therefore, we do not recommend arsenic trioxide as an anti-platelet agent against the hyperactive network state in ICU. Our simulations, on PGS activities, also indicated the potential efficacy of drugs like baricitinib in non-ICU and dasatinib in the ICU network, although their impact was not as pronounced as that of fostamatinib on simulated platelet protein activity.

Our simulations confirmed fostamatinib’s high potential to inhibit platelet hyperactivity compared to other highly ranked drugs. We confirm that fostamatinib can inhibit most of the platelet genes upregulated in ICU and non-ICU conditions. Our network shows the anti-platelet effects of fostamatinib via its targets in addition to SYK and we computationally and experimentally validated its inhibitory effect on SYK and SFKs. Its effects through SYK have been shown before as interfering with thrombosis but not with hemostasis (78, 79) and its potential use in COVID-19 has also been suggested by a computational study that confirmed fostamatinib’s targets by molecular docking (80). It was shown to counteract the platelet hyperactivity *in vitro* on platelets from COVID-19 patients with potentially actionable pathways as central for platelet activation and/or vascular complications (81). Moreover, there are clinical trials currently running on fostamatinib’s use in COVID-19 patients (Phase II: NCT04579393, NCT04581954; Phase III: NCT04629703 and NCT04924660).

### 4.4 Immunomodulation and fostamatinib: relevance in severe COVID-19 and vaccinated populations

In addressing severe COVID-19, there is a pressing need for immunomodulatory treatments that balance inflammation and antiviral responses (82). Severe cases linked to COVID-19-related coagulopathy, involve a dysregulated immune response, endothelial dysfunction, platelet hyperactivity, and impaired coagulation system, contributing to multiorgan dysfunction (82–84). SARS-CoV-2 spike-specific IgG antibodies contribute significantly to disease severity by triggering macrophage hyperactivation and thrombus formation (82). Small molecule inhibitors like SYK inhibitors show promise in countering anti-spike-IgG-induced inflammation and endothelial dysfunction. Notably, compounds targeting SYK and PI3K activity provide potential treatment avenues for severe COVID-19, specifically addressing inflammation induced by anti-spike immune complexes (82).

While our study primarily focuses on fostamatinib’s effect on platelet hyperactivity, it can also impact COVID-19 outcomes by addressing hyperinflammation in various immune cells (73). Fostamatinib’s inhibition of SYK plays a crucial role in reducing pro-inflammatory cytokine release, neutrophil extracellular trap production, and platelet aggregation, offering potential relief from organ dysfunction in critically ill COVID-19 patients (51, 85–88).

Considering our study’s use of data from 2020, predating widespread vaccination, it’s essential to explore two aspects to why our findings might remain relevant in a vaccinated population. First, even with fewer severe COVID-19 cases, the distinction between ICU and non-ICU patients remains vital (89), validating our drug rankings based on platelet network states. Our primary focus on the critical state of COVID-19, particularly in ICU patients, remains crucial, showing the consistent validity of our drug rankings based on platelet networks, even in severe cases, irrespective of vaccination status. This perspective, emphasizing the ongoing importance of addressing COVID-19 effects on platelets, holds true, especially considering observations of declining vaccine effectiveness (90–92). Second, our drug analysis may take on added significance, particularly concerning Vaccine-Induced Thrombotic Thrombocytopenia (VITT), where excessive platelet aggregation is observed via FcγRIIA-Syk signaling. Antiplatelet drugs tested by Smith et al. (2021) showed promise suppressing the platelet aggregation in response to patient sera in VITT. However, further studies evaluating clinical relevance are needed before considering these drugs. Some pose bleeding risks, but rilzabrutinib and fostamatinib show promise in addressing thrombosis without causing bleeding, although R406 demonstrated somewhat constrained effect *in vitro* (93–95). These findings, although needing additional validation, suggest potential applications in vaccinated populations, particularly in the context of VITT. This implies that our framework may be helpful not only against critical COVID-19 infections but against vaccine-induced thrombotic complications.

In conclusion, our study not only contributes insights into the treatment of severe COVID-19 cases but also prompts the need for expanded research encompassing various populations, including those vaccinated. Once available data on vaccinated populations emerge, our approach, based on molecular insights and platelet network states, is easily applicable. It is also transferrable to endothelial cells and leukocytes, representing the next logical steps in refining treatment strategies for a broader spectrum of treatment scenarios, from COVID-19 cases to other septic conditions with platelet hyperactivity.

These findings support growing interest in fostamatinib’s potential role in COVID-19 treatment, reinforcing the link between fostamatinib, platelet function, and COVID-19. This underscores fostamatinib’s potential in mitigating platelet hyperactivity, particularly in ICU patients, emphasizing the importance of considering network-wide effects when repurposing drugs for complex conditions such as COVID-19 or other severe septic conditions.

## Data availability statement

Publicly available sequencing datasets were analyzed in this study. This data can be found here: https://www.ncbi.nlm.nih.gov/bioproject/PRJNA634489/. The used data is referenced in the article. All other data and data-sets generated or analyzed during this study are included in this published article (and its **Supplementary Information Files**).

## Ethics statement

In this study, blood extraction from mice, which had not undergone any treatment prior to being sacrificed, was performed in compliance with the German Animal Welfare Act (TierSchG). Blood was immediately collected following euthanasia. This process did not require specific experimental approval.

## Author contributions

ÖO: Conceptualization, Formal analysis, Investigation, Writing – original draft, Writing – review & editing, Data curation, Methodology, Software, Visualization. SG: Conceptualization, Data curation, Formal analysis, Investigation, Methodology, Software, Visualization, Writing – original draft, Writing – review & editing, Supervision. AA: Data curation, Formal analysis, Investigation, Methodology, Visualization, Writing – review & editing. SY: Data curation, Formal analysis, Investigation, Methodology, Visualization, Writing – review & editing. MS: Formal analysis, Investigation, Methodology, Writing – review & editing. GA: Investigation, Methodology, Writing – review & editing, Validation. ZN: Investigation, Methodology, Validation, Writing – review & editing, Supervision. JB: Investigation, Methodology, Writing – review & editing, Data curation, Formal analysis, Software, Visualization. TD: Formal analysis, Investigation, Writing – review & editing, Conceptualization, Funding acquisition, Resources, Supervision, Writing – original draft.

## References

[1] Klok FA Kruip M van der Meer NJM Arbous MS Gommers D Kant KM. Confirmation of the high cumulative incidence of thrombotic complications in critically ill ICU patients with COVID-19: An updated analysis. Thromb Res (2020) 191:148–50. doi: 10.1016/j.thromres.2020.04.041 PMC7192101 32381264

[2] Wu C Chen X Cai Y Xia J Zhou X Xu S. Risk factors associated with acute respiratory distress syndrome and death in patients with coronavirus disease 2019 pneumonia in Wuhan, China. JAMA Intern Med (2020) 180(7):934–43. doi: 10.1001/jamainternmed.2020.0994 PMC7070509 32167524

[3] Guan WJ Ni ZY Hu Y Liang WH Ou CQ He JX. Clinical characteristics of coronavirus disease 2019 in China. N Engl J Med (2020) 382(18):1708–20. doi: 10.1056/NEJMoa2002032 PMC7092819 32109013

[4] Sciaudone A Corkrey H Humphries F Koupenova M. Platelets and SARS-CoV-2 during COVID-19: immunity, thrombosis, and beyond. Circ Res (2023) 132(10):1272–89. doi: 10.1161/CIRCRESAHA.122.321930 PMC10171305 37167360

[5] Andrews RK Berndt MC. Platelet physiology and thrombosis. Thromb Res (2004) 114(5-6):447–53. doi: 10.1016/j.thromres.2004.07.020 15507277

[6] Assinger A Kral JB Yaiw KC Schrottmaier WC Kurzejamska E Wang Y. Human cytomegalovirus-platelet interaction triggers toll-like receptor 2-dependent proinflammatory and proangiogenic responses. Arterioscler Thromb Vasc Biol (2014) 34(4):801–9. doi: 10.1161/ATVBAHA.114.303287 24558109

[7] Chaipan C Soilleux EJ Simpson P Hofmann H Gramberg T Marzi A. DC-SIGN and CLEC-2 mediate human immunodeficiency virus type 1 capture by platelets. J Virol (2006) 80(18):8951–60. doi: 10.1128/JVI.00136-06 PMC1563896 16940507

[8] Hottz ED Oliveira MF Nunes PC Nogueira RM Valls-de-Souza R Da Poian AT. Dengue induces platelet activation, mitochondrial dysfunction and cell death through mechanisms that involve DC-SIGN and caspases. J Thromb Haemost (2013) 11(5):951–62. doi: 10.1111/jth.12178 PMC3971842 23433144

[9] Hottz ED Lopes JF Freitas C Valls-de-Souza R Oliveira MF Bozza MT. Platelets mediate increased endothelium permeability in dengue through NLRP3-inflammasome activation. Blood (2013) 122(20):3405–14. doi: 10.1182/blood-2013-05-504449 PMC3829114 24009231

[10] Zahn A Jennings N Ouwehand WH Allain JP. Hepatitis C virus interacts with human platelet glycoprotein VI. J Gen Virol (2006) 87(Pt 8):2243–51. doi: 10.1099/vir.0.81826-0 16847120

[11] McElroy A. Understanding bleeding in ebola virus disease. Clin Adv Hematol Oncol (2015) 13(1):29–31. 25679971 PMC4667727

[12] Zapata JC Cox D Salvato MS. The role of platelets in the pathogenesis of viral hemorrhagic fevers. PloS Negl Trop Dis (2014) 8(6):e2858. doi: 10.1371/journal.pntd.0002858 24921924 PMC4055450

[13] Zhang S Liu Y Wang X Yang L Li H Wang Y. SARS-CoV-2 binds platelet ACE2 to enhance thrombosis in COVID-19. J Hematol Oncol (2020) 13(1):120. doi: 10.1186/s13045-020-00954-7 32887634 PMC7471641

[14] Poissy J Goutay J Caplan M Parmentier E Duburcq T Lassalle F. Pulmonary embolism in patients with COVID-19: awareness of an increased prevalence. Circulation (2020) 142(2):184–6. doi: 10.1161/CIRCULATIONAHA.120.047430 32330083

[15] Barrett TJ Lee AH Xia Y Lin LH Black M Cotzia P. Platelet and vascular biomarkers associate with thrombosis and death in coronavirus disease. Circ Res (2020) 127(7):945–7. doi: 10.1161/CIRCRESAHA.120.317803 PMC7478197 32757722

[16] Comer SP Cullivan S Szklanna PB Weiss L Cullen S Kelliher S. COVID-19 induces a hyperactive phenotype in circulating platelets. PloS Biol (2021) 19(2):e3001109. doi: 10.1371/journal.pbio.3001109 33596198 PMC7920383

[17] Shen S Zhang J Fang Y Lu S Wu J Zheng X. SARS-CoV-2 interacts with platelets and megakaryocytes *via* ACE2-independent mechanism. J Hematol Oncol (2021) 14(1):72. doi: 10.1186/s13045-021-01082-6 33926500 PMC8082485

[18] Nicolai L Leunig A Brambs S Kaiser R Weinberger T Weigand M. Immunothrombotic dysregulation in COVID-19 pneumonia is associated with respiratory failure and coagulopathy. Circulation (2020) 142(12):1176–89. doi: 10.1161/CIRCULATIONAHA.120.048488 PMC7497892 32755393

[19] Manne BK Denorme F Middleton EA Portier I Rowley JW Stubben C. Platelet gene expression and function in patients with COVID-19. Blood (2020) 136(11):1317–29. doi: 10.1182/blood.2020007214 PMC7483430 32573711

[20] Schrottmaier WC Pirabe A Pereyra D Heber S Hackl H Schmuckenschlager A. Platelets and antiplatelet medication in COVID-19-related thrombotic complications. Front Cardiovasc Med (2021) 8:802566. doi: 10.3389/fcvm.2021.802566 35141292 PMC8818754

[21] Weiss LJ Drayss M Manukjan G Zeitlhofler M Kleiss J Weigel M. Uncoupling of platelet granule release and integrin activation suggests GPIIb/IIIa as a therapeutic target in COVID-19. Blood Adv (2023) 7(11):2324–38. doi: 10.1182/bloodadvances.2022008666 PMC9462922 36053793

[22] Denorme F Manne BK Portier I Petrey AC Middleton EA Kile BT. COVID-19 patients exhibit reduced procoagulant platelet responses. J Thromb Haemost (2020) 18(11):3067–73. doi: 10.1111/jth.15107 PMC7646270 32945081

[23] Li M Gao H Wang J Wu FX. Control principles for complex biological networks. Brief Bioinform (2019) 20(6):2253–66. doi: 10.1093/bib/bby088 30239577

[24] Wang LZ Chen YZ Wang WX Lai YC. Physical controllability of complex networks. Sci Rep (2017) 7:40198. doi: 10.1038/srep40198 28074900 PMC5225471

[25] Vinayagam A Gibson TE Lee HJ Yilmazel B Roesel C Hu Y. Controllability analysis of the directed human protein interaction network identifies disease genes and drug targets. Proc Natl Acad Sci U.S.A. (2016) 113(18):4976–81. doi: 10.1073/pnas.1603992113 PMC4983807 27091990

[26] Jia T Liu YY Csoka E Posfai M Slotine JJ Barabasi AL. Emergence of bimodality in controlling complex networks. Nat Commun (2013) 4:2002. doi: 10.1038/ncomms3002 23774965

[27] Wishart DS Feunang YD Guo AC Lo EJ Marcu A Grant JR. DrugBank 5.0: a major update to the DrugBank database for 2018. Nucleic Acids Res (2018) 46(D1):D1074–D82. doi: 10.1093/nar/gkx1037 PMC5753335 29126136

[28] Wingett SW Andrews S. FastQ Screen: A tool for multi-genome mapping and quality control. F1000Res (2018) 7:1338. doi: 10.12688/f1000research.15931.1 30254741 PMC6124377

[29] Dobin A Davis CA Schlesinger F Drenkow J Zaleski C Jha S. STAR: ultrafast universal RNA-seq aligner. Bioinformatics (2013) 29(1):15–21. doi: 10.1093/bioinformatics/bts635 23104886 PMC3530905

[30] Liao Y Smyth GK Shi W. featureCounts: an efficient general purpose program for assigning sequence reads to genomic features. Bioinformatics (2014) 30(7):923–30. doi: 10.1093/bioinformatics/btt656 24227677

[31] Love MI Huber W Anders S. Moderated estimation of fold change and dispersion for RNA-seq data with DESeq2. Genome Biol (2014) 15(12):550. doi: 10.1186/s13059-014-0550-8 25516281 PMC4302049

[32] Gene Ontology C. The Gene Ontology resource: enriching a GOld mine. Nucleic Acids Res (2021) 49(D1):D325–D34. doi: 10.1093/nar/gkaa1113 PMC7779012 33290552

[33] Kabir MH Patrick R Ho JWK O'Connor MD. Identification of active signaling pathways by integrating gene expression and protein interaction data. BMC Syst Biol (2018) 12(Suppl 9):120. doi: 10.1186/s12918-018-0655-x 30598083 PMC6311899

[34] Treveil A Bohar B Sudhakar P Gul L Csabai L Olbei M. ViralLink: An integrated workflow to investigate the effect of SARS-CoV-2 on intracellular signalling and regulatory pathways. PloS Comput Biol (2021) 17(2):e1008685. doi: 10.1371/journal.pcbi.1008685 33534793 PMC7886129

[35] Shannon P Markiel A Ozier O Baliga NS Wang JT Ramage D. Cytoscape: a software environment for integrated models of biomolecular interaction networks. Genome Res (2003) 13(11):2498–504. doi: 10.1101/gr.1239303 PMC403769 14597658

[36] Buijsers B Yanginlar C de Nooijer A Grondman I Maciej-Hulme ML Jonkman I. Increased plasma heparanase activity in COVID-19 patients. Front Immunol (2020) 11:575047. doi: 10.3389/fimmu.2020.575047 33123154 PMC7573491

[37] Eustes AS Campbell RA Middleton EA Tolley ND Manne BK Montenont E. Heparanase expression and activity are increased in platelets during clinical sepsis. J Thromb Haemost (2021) 19(5):1319–30. doi: 10.1111/jth.15266 PMC8218538 33587773

[38] Ogishima T Shiina H Breault JE Terashima M Honda S Enokida H. Promoter CpG hypomethylation and transcription factor EGR1 hyperactivate heparanase expression in bladder cancer. Oncogene (2005) 24(45):6765–72. doi: 10.1038/sj.onc.1208811 16007175

[39] Hadigal SR Agelidis AM Karasneh GA Antoine TE Yakoub AM Ramani VC. Heparanase is a host enzyme required for herpes simplex virus-1 release from cells. Nat Commun (2015) 6:6985. doi: 10.1038/ncomms7985 25912399 PMC4413471

[40] Nacher JC Ishitsuka M Miyazaki S Akutsu T. Finding and analysing the minimum set of driver nodes required to control multilayer networks. Sci Rep (2019) 9(1):576. doi: 10.1038/s41598-018-37046-z 30679639 PMC6345816

[41] Molnár F Sreenivasan S Syzmanski BK Korniss G. Minimum dominating sets in scale-free network ensembles. Sci Rep (2013) 2013(3):1736. doi: 10.1038/srep01736

[42] Wu L Li M Wang J Wu FX. CytoCtrlAnalyser: a Cytoscape app for biomolecular network controllability analysis. Bioinformatics (2018) 34(8):1428–30. doi: 10.1093/bioinformatics/btx764 29186337

[43] Nacher JC Akutsu T. Structural controllability of unidirectional bipartite networks. Sci Rep (2013) 3:1647. doi: 10.1038/srep01647 23571689 PMC3622082

[44] Bikdeli B Madhavan MV Gupta A Jimenez D Burton JR Der Nigoghossian C. Pharmacological agents targeting thromboinflammation in COVID-19: review and implications for future research. Thromb Haemost (2020) 120(7):1004–24. doi: 10.1055/s-0040-1713152 PMC7516364 32473596

[45] Gil DP Law JN Murali TM. The PathLinker app: Connect the dots in protein interaction networks. F1000Res (2017) 6:58. doi: 10.12688/f1000research.9909.1 28413614 PMC5365231

[46] Wool GD Miller JL. The impact of COVID-19 disease on platelets and coagulation. Pathobiology (2021) 88(1):15–27. doi: 10.1159/000512007 33049751 PMC7649697

[47] Hottz ED Azevedo-Quintanilha IG Palhinha L Teixeira L Barreto EA Pao CRR. Platelet activation and platelet-monocyte aggregate formation trigger tissue factor expression in patients with severe COVID-19. Blood (2020) 136(11):1330–41. doi: 10.1182/blood.2020007252 PMC7483437 32678428

[48] Ravindran V Nacher JC Akutsu T Ishitsuka M Osadcenco A Sunitha V. Network controllability analysis of intracellular signalling reveals viruses are actively controlling molecular systems. Sci Rep (2019) 9(1):2066. doi: 10.1038/s41598-018-38224-9 30765882 PMC6375943

[49] Liu YY Slotine JJ Barabasi AL. Controllability of complex networks. Nature (2011) 473(7346):167–73. doi: 10.1038/nature10011 21562557

[50] Liu YY Slotine JJ Barabasi AL. Control centrality and hierarchical structure in complex networks. PloS One (2012) 7(9):e44459. doi: 10.1371/journal.pone.0044459 23028542 PMC3459977

[51] Strich JR Ramos-Benitez MJ Randazzo D Stein SR Babyak A Davey RT. Fostamatinib inhibits neutrophils extracellular traps induced by COVID-19 patient plasma: A potential therapeutic. J Infect Dis (2021) 223(6):981–4. doi: 10.1093/infdis/jiaa789 PMC7799006 33367731

[52] Connell NT Berliner N. Fostamatinib for the treatment of chronic immune thrombocytopenia. Blood (2019) 133(19):2027–30. doi: 10.1182/blood-2018-11-852491 30803989

[53] Spalton JC Mori J Pollitt AY Hughes CE Eble JA Watson SP. The novel Syk inhibitor R406 reveals mechanistic differences in the initiation of GPVI and CLEC-2 signaling in platelets. J Thromb Haemost (2009) 7(7):1192–9. doi: 10.1111/j.1538-7836.2009.03451.x 19422460

[54] Ninomoto J Mokatrin A Kinoshita T Marimpietri C Barrett TD Chang BY. Effects of ibrutinib on in *vitro* platelet aggregation in blood samples from healthy donors and donors with platelet dysfunction. Hematology (2020) 25(1):112–7. doi: 10.1080/16078454.2020.1730080 32131714

[55] Cines DB Greinacher A. Vaccine-induced immune thrombotic thrombocytopenia. Blood (2023) 141(14):1659–65. doi: 10.1182/blood.2022017696 PMC9870607 36669155

[56] Edmonds R Schonborn L Habben S Paparoupa M Greinacher A Schuppert F. Vaccine-induced immune thrombotic thrombocytopenia (VITT) after SARS-CoV-2 vaccination: Two cases from Germany with unusual presentation. Clin Case Rep (2023) 11(1):e6883. doi: 10.1002/ccr3.6883 36698527 PMC9859986

[57] Liu X Hong Z Liu J Lin Y Rodriguez-Paton A Zou Q. Computational methods for identifying the critical nodes in biological networks. Brief Bioinform (2020) 21(2):486–97. doi: 10.1093/bib/bbz011 30753282

[58] Rolf MG Curwen JO Veldman-Jones M Eberlein C Wang J Harmer A. *In vitro* pharmacological profiling of R406 identifies molecular targets underlying the clinical effects of fostamatinib. Pharmacol Res Perspect (2015) 3(5):e00175. doi: 10.1002/prp2.175 26516587 PMC4618646

[59] Vidal M Cusick ME Barabasi AL. Interactome networks and human disease. Cell (2011) 144(6):986–98. doi: 10.1016/j.cell.2011.02.016 PMC3102045 21414488

[60] Durmus S Cakir T Ozgur A Guthke R. A review on computational systems biology of pathogen-host interactions. Front Microbiol (2015) 6:235. doi: 10.3389/fmicb.2015.00235 25914674 PMC4391036

[61] Schenone M Dancik V Wagner BK Clemons PA. Target identification and mechanism of action in chemical biology and drug discovery. Nat Chem Biol (2013) 9(4):232–40. doi: 10.1038/nchembio.1199 PMC5543995 23508189

[62] Xie L Li J Xie L Bourne PE. Drug discovery using chemical systems biology: identification of the protein-ligand binding network to explain the side effects of CETP inhibitors. PloS Comput Biol (2009) 5(5):e1000387. doi: 10.1371/journal.pcbi.1000387 19436720 PMC2676506

[63] Parvathaneni V Kulkarni NS Muth A Gupta V. Drug repurposing: a promising tool to accelerate the drug discovery process. Drug Discovery Today (2019) 24(10):2076–85. doi: 10.1016/j.drudis.2019.06.014 PMC11920972 31238113

[64] Cheng F Desai RJ Handy DE Wang R Schneeweiss S Barabasi AL. Network-based approach to prediction and population-based validation of in silico drug repurposing. Nat Commun (2018) 9(1):2691. doi: 10.1038/s41467-018-05116-5 30002366 PMC6043492

[65] Jang WD Jeon S Kim S Lee SY. Drugs repurposed for COVID-19 by virtual screening of 6,218 drugs and cell-based assay. Proc Natl Acad Sci U.S.A. (2021) 118(30):e2024302118. doi: 10.1073/pnas.2024302118 34234012 PMC8325362

[66] Gordon DE Jang GM Bouhaddou M Xu J Obernier K White KM. A SARS-CoV-2 protein interaction map reveals targets for drug repurposing. Nature (2020) 583(7816):459–68. doi: 10.1038/s41586-020-2286-9 PMC7431030 32353859

[67] Han N Hwang W Tzelepis K Schmerer P Yankova E MacMahon M. Identification of SARS-CoV-2-induced pathways reveals drug repurposing strategies. Sci Adv (2021) 7(27):eabh3032. doi: 10.1126/sciadv.abh3032 34193418 PMC8245040

[68] Zhou Y Hou Y Shen J Huang Y Martin W Cheng F. Network-based drug repurposing for novel coronavirus 2019-nCoV/SARS-CoV-2. Cell Discovery (2020) 6:14. doi: 10.1038/s41421-020-0153-3 32194980 PMC7073332

[69] Bakowski MA Beutler N Wolff KC Kirkpatrick MG Chen E Nguyen TH. Drug repurposing screens identify chemical entities for the development of COVID-19 interventions. Nat Commun (2021) 12(1):3309. doi: 10.1038/s41467-021-23328-0 34083527 PMC8175350

[70] Fiscon G Conte F Farina L Paci P. SAveRUNNER: A network-based algorithm for drug repurposing and its application to COVID-19. PloS Comput Biol (2021) 17(2):e1008686. doi: 10.1371/journal.pcbi.1008686 33544720 PMC7891752

[71] Siminea N Popescu V Sanchez Martin JA Florea D Gavril G Gheorghe AM. Network analytics for drug repurposing in COVID-19. Brief Bioinform (2022) 23(1):bbab490. doi: 10.1093/bib/bbab490 34864885 PMC8690228

[72] Wei X Pan C Zhang X Zhang W. Total controllability analysis discovers explainable drugs for Covid-19 treatment. Biol Direct (2023) 18(1):55. doi: 10.21203/rs.3.rs-3147521/v1 37670359 PMC10478273

[73] Gupta SK Srivastava M Minocha R Akash A Dangwal S Dandekar T. Alveolar regeneration in COVID-19 patients: A network perspective. Int J Mol Sci (2021) 22(20):11279. doi: 10.3390/ijms222011279 34681944 PMC8538208

[74] Braselmann S Taylor V Zhao H Wang S Sylvain C Baluom M. R406, an orally available spleen tyrosine kinase inhibitor blocks fc receptor signaling and reduces immune complex-mediated inflammation. J Pharmacol Exp Ther (2006) 319(3):998–1008. doi: 10.1124/jpet.106.109058 16946104

[75] Mocsai A Ruland J Tybulewicz VL. The SYK tyrosine kinase: a crucial player in diverse biological functions. Nat Rev Immunol (2010) 10(6):387–402. doi: 10.1038/nri2765 20467426 PMC4782221

[76] Rojewski MT Baldus C Knauf W Thiel E Schrezenmeier H. Dual effects of arsenic trioxide (As2O3) on non-acute promyelocytic leukaemia myeloid cell lines: induction of apoptosis and inhibition of proliferation. Br J Haematol (2002) 116(3):555–63. doi: 10.1046/j.0007-1048.2001.03298.x 11849211

[77] Lin KH Chang YF Fan CY Jayakumar T Lee JJ Chou DS. Arsenic trioxide-mediated antiplatelet activity: pivotal role of the phospholipase C gamma 2-protein kinase C-p38 MAPK cascade. Transl Res (2010) 155(2):97–108. doi: 10.1016/j.trsl.2009.08.005 20129490

[78] Mehta AR Kefela A Toste C Sweet D. Real-world use of fostamatinib in patients with immune thrombocytopenia and thrombotic risk. Acta Haematol (2022) 145(2):221–8. doi: 10.1159/000520438 PMC9116595 34913873

[79] Harbi MH Smith CW Alenazy FO Nicolson PLR Tiwari A Watson SP. Antithrombotic effects of fostamatinib in combination with conventional antiplatelet drugs. Int J Mol Sci (2022) 23(13):6982. doi: 10.3390/ijms23136982 35805988 PMC9266367

[80] Saha S Halder AK Bandyopadhyay SS Chatterjee P Nasipuri M Bose D. Drug repurposing for COVID-19 using computational screening: Is Fostamatinib/R406 a potential candidate? Methods (2022) 203:564–74. doi: 10.1016/j.ymeth.2021.08.007 PMC8390099 34455072

[81] Apostolidis SA Sarkar A Giannini HM Goel RR Mathew D Suzuki A. Signaling through FcgammaRIIA and the C5a-C5aR pathway mediate platelet hyperactivation in COVID-19. Front Immunol (2022) 13:834988. doi: 10.3389/fimmu.2022.834988 35309299 PMC8928747

[82] Geyer CE Chen HJ Bye AP Manz XD Guerra D Caniels TG. Identification of new drugs to counteract anti-spike IgG-induced hyperinflammation in severe COVID-19. Life Sci Alliance (2023) 6(11):e202302106. doi: 10.26508/lsa.202302106 37699657 PMC10497933

[83] Shankar A Varadan B Ethiraj D Sudarsanam H Hakeem AR Kalyanasundaram S. Systemic arterio-venous thrombosis in COVID-19: A pictorial review. World J Radiol (2021) 13(1):19–28. doi: 10.4329/wjr.v13.i1.19 33574991 PMC7852348

[84] Higashikuni Y Liu W Obana T Sata M. Pathogenic basis of thromboinflammation and endothelial injury in COVID-19: current findings and therapeutic implications. Int J Mol Sci (2021) 22(21):12081. doi: 10.3390/ijms222112081 34769508 PMC8584434

[85] Hoepel W Chen HJ Geyer CE Allahverdiyeva S Manz XD de Taeye SW. High titers and low fucosylation of early human anti-SARS-CoV-2 IgG promote inflammation by alveolar macrophages. Sci Transl Med (2021) 13(596):eabf8654. doi: 10.1126/scitranslmed.abf8654 33979301 PMC8158960

[86] Sung PS Hsieh SL. CLEC2 and CLEC5A: pathogenic host factors in acute viral infections. Front Immunol (2019) 10:2867. doi: 10.3389/fimmu.2019.02867 31867016 PMC6909378

[87] Bye AP Hoepel W Mitchell JL Jegouic S Loureiro S Sage T. Aberrant glycosylation of anti-SARS-CoV-2 spike IgG is a prothrombotic stimulus for platelets. Blood (2021) 138(16):1481–9. doi: 10.1182/blood.2021011871 PMC8321687 34315173

[88] Wigerblad G Warner SA Ramos-Benitez MJ Kardava L Tian X Miao R. Spleen tyrosine kinase inhibition restores myeloid homeostasis in COVID-19. Sci Adv (2023) 9(1):eade8272. doi: 10.1126/sciadv.ade8272 36598976 PMC9812373

[89] van Diepen S McAlister FA Chu LM Youngson E Kaul P Kadri SS. Association between vaccination status and outcomes in patients admitted to the ICU with COVID-19. Crit Care Med (2023) 51(9):1201–9. doi: 10.1097/CCM.0000000000005928 37192450

[90] Andrews N Stowe J Kirsebom F Toffa S Rickeard T Gallagher E. Covid-19 vaccine effectiveness against the omicron (B.1.1.529) variant. N Engl J Med (2022) 386(16):1532–46. doi: 10.1056/NEJMoa2119451 PMC8908811 35249272

[91] Feikin DR Higdon MM Abu-Raddad LJ Andrews N Araos R Goldberg Y. Duration of effectiveness of vaccines against SARS-CoV-2 infection and COVID-19 disease: results of a systematic review and meta-regression. Lancet (2022) 399(10328):924–44. doi: 10.1016/S0140-6736(22)00152-0 PMC8863502 35202601

[92] Planas D Saunders N Maes P Guivel-Benhassine F Planchais C Buchrieser J. Considerable escape of SARS-CoV-2 Omicron to antibody neutralization. Nature (2022) 602(7898):671–5. doi: 10.1038/s41586-021-04389-z 35016199

[93] Smith CW Montague SJ Kardeby C Di Y Lowe GC Lester WA. Antiplatelet drugs block platelet activation by VITT patient serum. Blood (2021) 138(25):2733–40. doi: 10.1182/blood.2021012277 PMC8697531 34375398

[94] Strich JR Kanthi Y. VITT(al) insights into vaccine-related clots. Blood (2021) 138(22):2159–60. doi: 10.1182/blood.2021014195 PMC8638213 34854882

[95] Greinacher A Selleng K Palankar R Wesche J Handtke S Wolff M. Insights in ChAdOx1 nCoV-19 vaccine-induced immune thrombotic thrombocytopenia. Blood (2021) 138(22):2256–68. doi: 10.1182/blood.2021013231 PMC8483989 34587242

