# Supplementary material for: Signaling network analysis reveals fostamatinib as a potential drug to control platelet hyperactivation during SARS-CoV-2 infection
Source: Front Immunol. 2023 Dec 21;14:1285345. doi: 10.3389/fimmu.2023.1285345 (PMC10768010; doi:10.3389/fimmu.2023.1285345)
Supplement: Supplementary file 2 [file DataSheet_1.docx]

# **Supplementary Notes**

## Background to network-building methods

To get reliable networks, we used two different network-building methods (SPAGI and ViralLink). SPAGI (Kabir et al., 2018) identifies the potentially active signaling pathways for a given set of proteins and transcriptome data. SPAGI models the network as a directed weighted graph, where the potential signaling pathways consist of proteins according to the input transcriptome data, and subsequently, the active pathways are identified based on the proportion of highly expressed genes. Given a set of genes as input, SPAGI can model both the active and likely active signaling pathways. ViralLink (Treveil et al., 2021) also identifies the signaling network based on transcriptomic data but can also identify the nodes interacting with SARS-CoV-2. The difference between the two is that SPAGI uses directed prior protein-protein interaction data in the background and identifies short paths from the receptors to the transcription factors, while ViralLink uses known signaling networks and identifies comparative longer pathways from viral proteins to differentially expressed genes downstream to transcription factors. The power of both programs are used to merge reconstructed networks.

## Previous drug repurposing studies

Besides understanding the interactome (Vidal et al., 2011), and pathogen-host interactions (Durmus et al., 2015), network-based methods have traditionally been used for investigating drug mechanisms (Schenone et al., 2013), discovering potential side-effects (Xie et al., 2009), searching for novel drug targets, or drug repurposing (Parvathaneni et al., 2019). In such a pandemic, drug repurposing is especially important because it is time- and cost-efficient as opposed to new drug discovery. For instance, Cheng et al. created a human protein-protein interaction network (Cheng et al., 2018; Parvathaneni et al., 2019) , with which they could reveal drug-disease indications, undesirable side effects, and potential mechanisms of the actions of drugs. The network showed over 70% accuracy for the identification of well-known drug indications by quantifying the network proximity of disease genes and drug targets. It was used to show the mechanisms of cardiovascular drugs and was able to reveal their side effects, which were validated with a large-scale patient cohort. Recently, several network-based drug repurposing studies have been published for SARS-CoV-2 (Bakowski et al., 2021; Gordon et al., 2020; Han et al., 2021; Jang et al., 2021; Zhou et al., 2020) however, none of them have focused on platelets. Gordon et al. identified SARS-CoV-2-human protein-protein interactions via affinity-purification mass spectrometry and screened these proteins for potential drug targets (Gordon et al., 2020). They found 69 ligands, including 29 FDA-approved drugs. Screening these drugs identified two sets of active drugs and compounds: those affecting translation and those modulating Sigma1 and Sigma2 receptors. In another study, multiple computational approaches, such as data integration, network analysis, computer simulation, and machine learning were used to identify 200 drugs that target SARS-CoV-2-induced pathways (Han et al., 2021). Among 200 drugs, 40 were already in COVID-19 clinical trials. Drug repurposing with a novel virtual screening strategy that reduces false positives were used against COVID-19 (Jang et al., 2021). It showed that three drug combinations could be effective against COVID-19, as they have antiviral properties with reduced drug toxicity. Zhou et al. used network proximity analysis between drug targets and human coronavirus-associated proteins to screen for candidate repurposable drugs for human coronaviruses (Zhou et al., 2020). They found 16 potential drugs that are further validated by enrichment analyses of drug-gene signatures and human coronavirus-induced transcriptomics data in human cell lines.

Because of the interactions between the genes/proteins, changes in a few nodes in a network can affect the whole system (Liu et al., 2020), but not all the nodes in the networks do not have similar importance for the integrity of the system. We first focused on signaling network building, as discussed above, then obtained the network node importance based on controllability analysis. Control theory has shown the potential to find the fewest nodes in the system, which can be controlled to drive the network from the current state to the desired state. Driver nodes, which are imposed by different signals, offer full controllability over a network (Liu et al., 2020). Indispensable nodes are crucial for the system as their removal increases the minimum number of driver nodes (size of MDS) to control the network. In other words, the removal of indispensable nodes reduces the control efficiency since more driver nodes need to be modulated to change the state of the network. Other important nodes in the system are critical nodes (indegree 0) which are present in every alternative MDS of the system.

**References:**

Bakowski, M. A., Beutler, N., Wolff, K. C., Kirkpatrick, M. G., Chen, E., Nguyen, T. H., Riva, L., Shaabani, N., Parren, M., Ricketts, J., Gupta, A. K., Pan, K., Kuo, P., Fuller, M., Garcia, E., Teijaro, J. R., Yang, L., Sahoo, D., Chi, V., . . . Rogers, T. F. (2021). Drug repurposing screens identify chemical entities for the development of COVID-19 interventions. *Nat Commun*, *12*(1), 3309. <https://doi.org/10.1038/s41467-021-23328-0>

Cheng, F., Desai, R. J., Handy, D. E., Wang, R., Schneeweiss, S., Barabasi, A. L., & Loscalzo, J. (2018). Network-based approach to prediction and population-based validation of in silico drug repurposing. *Nat Commun*, *9*(1), 2691. <https://doi.org/10.1038/s41467-018-05116-5>

Durmus, S., Cakir, T., Ozgur, A., & Guthke, R. (2015). A review on computational systems biology of pathogen-host interactions. *Front Microbiol*, *6*, 235. <https://doi.org/10.3389/fmicb.2015.00235>

Gordon, D. E., Jang, G. M., Bouhaddou, M., Xu, J., Obernier, K., White, K. M., O'Meara, M. J., Rezelj, V. V., Guo, J. Z., Swaney, D. L., Tummino, T. A., Huttenhain, R., Kaake, R. M., Richards, A. L., Tutuncuoglu, B., Foussard, H., Batra, J., Haas, K., Modak, M., . . . Krogan, N. J. (2020). A SARS-CoV-2 protein interaction map reveals targets for drug repurposing. *Nature*, *583*(7816), 459-468. <https://doi.org/10.1038/s41586-020-2286-9>

Han, N., Hwang, W., Tzelepis, K., Schmerer, P., Yankova, E., MacMahon, M., Lei, W., N, M. K., Liu, A., Felgenhauer, U., Schuldt, A., Harris, R., Chapman, K., McCaughan, F., Weber, F., & Kouzarides, T. (2021). Identification of SARS-CoV-2-induced pathways reveals drug repurposing strategies. *Sci Adv*, *7*(27). <https://doi.org/10.1126/sciadv.abh3032>

Jang, W. D., Jeon, S., Kim, S., & Lee, S. Y. (2021). Drugs repurposed for COVID-19 by virtual screening of 6,218 drugs and cell-based assay. *Proc Natl Acad Sci U S A*, *118*(30). <https://doi.org/10.1073/pnas.2024302118>

Kabir, M. H., Patrick, R., Ho, J. W. K., & O'Connor, M. D. (2018). Identification of active signaling pathways by integrating gene expression and protein interaction data. *BMC Syst Biol*, *12*(Suppl 9), 120. <https://doi.org/10.1186/s12918-018-0655-x>

Liu, X., Hong, Z., Liu, J., Lin, Y., Rodriguez-Paton, A., Zou, Q., & Zeng, X. (2020). Computational methods for identifying the critical nodes in biological networks. *Brief Bioinform*, *21*(2), 486-497. <https://doi.org/10.1093/bib/bbz011>

Parvathaneni, V., Kulkarni, N. S., Muth, A., & Gupta, V. (2019). Drug repurposing: a promising tool to accelerate the drug discovery process. *Drug Discov Today*, *24*(10), 2076-2085. <https://doi.org/10.1016/j.drudis.2019.06.014>

Schenone, M., Dancik, V., Wagner, B. K., & Clemons, P. A. (2013). Target identification and mechanism of action in chemical biology and drug discovery. *Nat Chem Biol*, *9*(4), 232-240. <https://doi.org/10.1038/nchembio.1199>

Treveil, A., Bohar, B., Sudhakar, P., Gul, L., Csabai, L., Olbei, M., Poletti, M., Madgwick, M., Andrighetti, T., Hautefort, I., Modos, D., & Korcsmaros, T. (2021). ViralLink: An integrated workflow to investigate the effect of SARS-CoV-2 on intracellular signalling and regulatory pathways. *PLoS Comput Biol*, *17*(2), e1008685. <https://doi.org/10.1371/journal.pcbi.1008685>

Vidal, M., Cusick, M. E., & Barabasi, A. L. (2011). Interactome networks and human disease. *Cell*, *144*(6), 986-998. <https://doi.org/10.1016/j.cell.2011.02.016>

Xie, L., Li, J., Xie, L., & Bourne, P. E. (2009). Drug discovery using chemical systems biology: identification of the protein-ligand binding network to explain the side effects of CETP inhibitors. *PLoS Comput Biol*, *5*(5), e1000387. <https://doi.org/10.1371/journal.pcbi.1000387>

Zhou, Y., Hou, Y., Shen, J., Huang, Y., Martin, W., & Cheng, F. (2020). Network-based drug repurposing for novel coronavirus 2019-nCoV/SARS-CoV-2. *Cell Discov*, *6*, 14. <https://doi.org/10.1038/s41421-020-0153-3>

# **Supplementary Figures**

**Figure S1**: **Comparison of non-ICU and ICU networks.** Number of interactions (edges) (A) and genes (nodes) (B) in ICU and non-ICU networks.


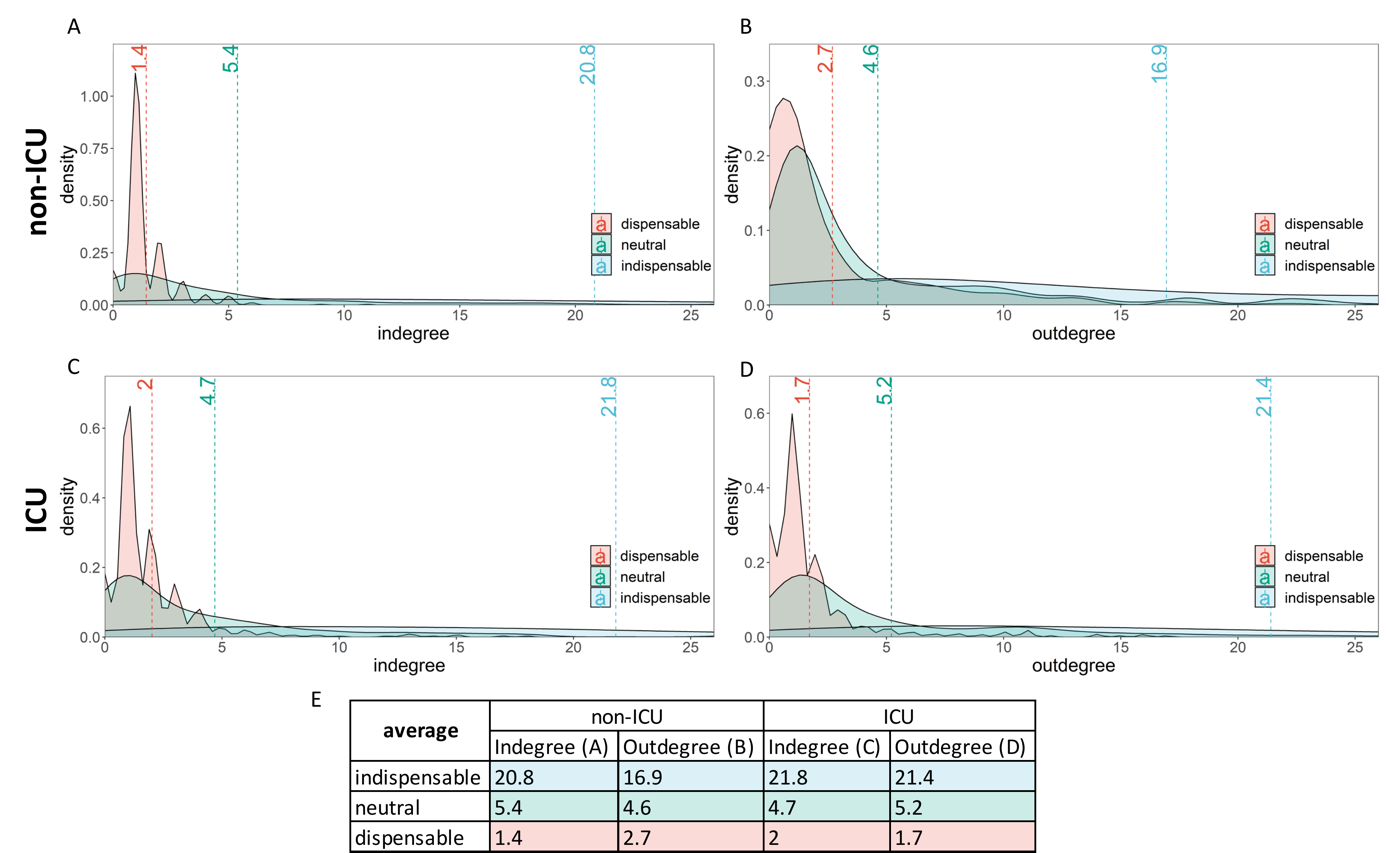


**Figure S2:** **Controllability in platelets comparing different types of nodes.** The hyperreactive platelets need key signaling nodes (indispensable nodes) as only those are well connected. This is true under both conditions. Distribution of the indegrees of indispensable (blue), neutral (green), and dispensable (red) nodes in (A) non-ICU (C) in ICU network and distribution of outdegrees in (B) non-ICU and (D) in ICU networks. (E) Table summarizing average in- and outdegree values for each category. Dotted lines represent the average in- and outdegree for each category, summarized in the table below. There are clear differences between both patient groups for the parameters analyzed as indicated. These point to less control within the platelets of the ICU patients (details see text).


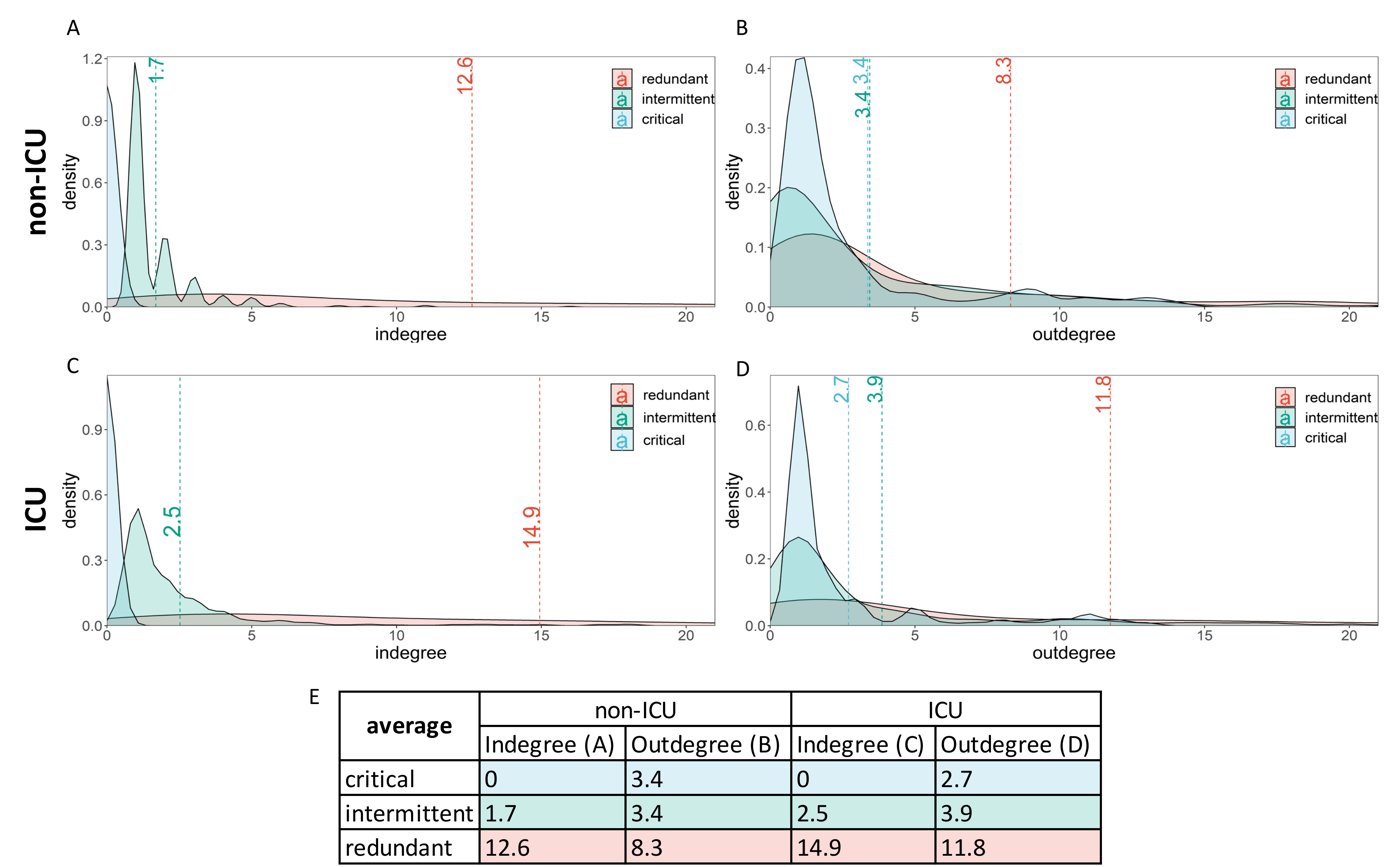


**Figure S3:** **Controllability in platelets comparing different types of nodes.** Distribution of the indegrees and outdegrees of redundant (red), intermittent (green), and critical (blue) nodes in (A) non-ICU (C) ICU networks and distribution of outdegrees in (B) non-ICU and (D) in ICU networks. (E) Table summarizing average in- and outdegree values for each category. Dotted lines represent the average in- and outdegree for each category, summarized in the table below.


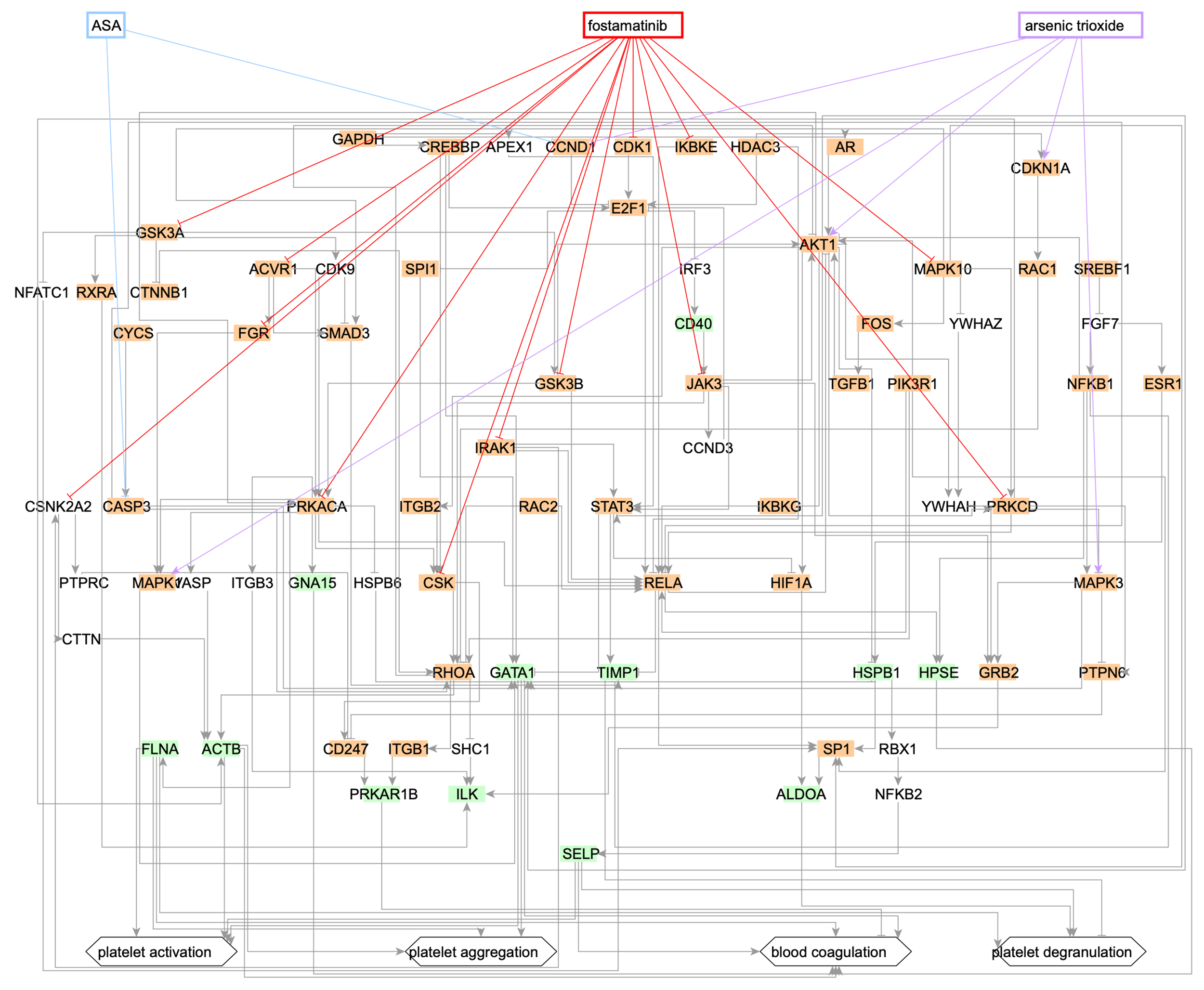


**Figure S4:** **Network state and simulations for ICU patients.** ICU network built by identifying one-to-one the shortest pathways between the filt-ind nodes and the platelet genes (upper panel) and simulation of the effects of the top 3 drugs on platelet genes (lower panel).


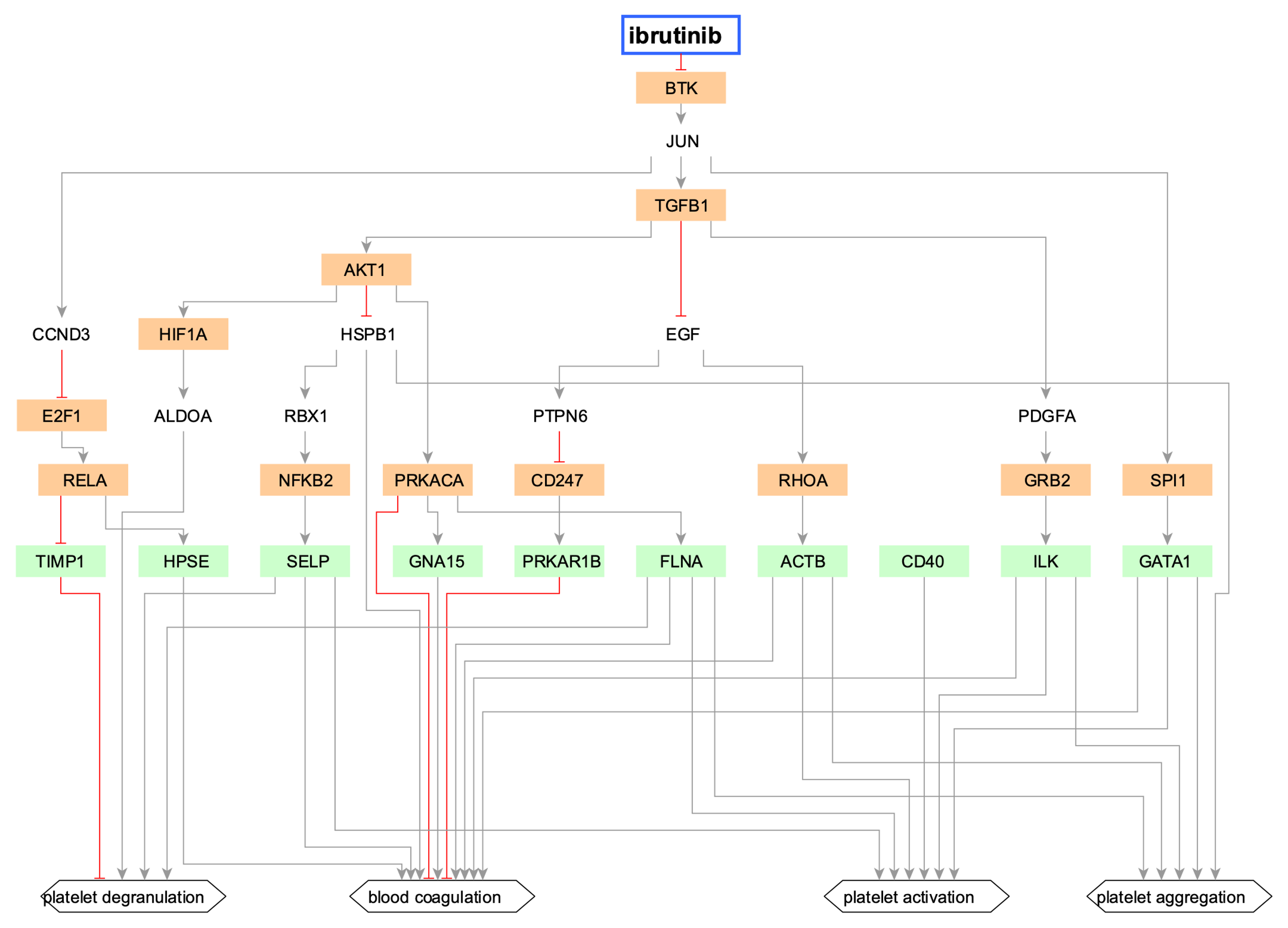


**Figure S5:** **Subnetwork that connects Ibrutinib’s target BTK** (Bruton tyrosine kinase) to platelet proteins in the ICU network (upper panel), simulations show the changes in platelet genes upon Ibrutinib activation (lower panel).
